# Differential root transcriptomics in a polyploid non-model crop: the importance of respiration during osmotic stress

**DOI:** 10.1038/srep22583

**Published:** 2016-03-03

**Authors:** Yasmín Zorrilla-Fontanesi, Mathieu Rouard, Alberto Cenci, Ewaut Kissel, Hien Do, Emeric Dubois, Sabine Nidelet, Nicolas Roux, Rony Swennen, Sebastien Christian Carpentier

**Affiliations:** 1 KU Leuven, Division of Crop Biotechnics, Laboratory of Tropical Crop Improvement, B-3001 Leuven, Belgium; 2 Bioversity International, Parc Scientifique Agropolis II, 34397 Montpellier Cedex 05, France; 3 MGX-Montpellier GenomiX, Montpellier Genomics and Bioinformatics Facility, Montpellier F-34396, France; 4 Bioversity International, Willem De Croylaan 42, B-3001 Leuven, Belgium; 5 International Institute of Tropical Agriculture. c/o AVRDC - The World Vegetable Center. P.O. Box 10, Duluti, Arusha, Tanzania

## Abstract

To explore the transcriptomic global response to osmotic stress in roots, 18 mRNA-seq libraries were generated from three triploid banana genotypes grown under mild osmotic stress (5% PEG) and control conditions. Illumina sequencing produced 568 million high quality reads, of which 70–84% were mapped to the banana diploid reference genome. Using different uni- and multivariate statistics, 92 genes were commonly identified as differentially expressed in the three genotypes. Using our in house workflow to analyze GO enriched and underlying biochemical pathways, we present the general processes affected by mild osmotic stress in the root and focus subsequently on the most significantly overrepresented classes associated with: respiration, glycolysis and fermentation. We hypothesize that in fast growing and oxygen demanding tissues, mild osmotic stress leads to a lower energy level, which induces a metabolic shift towards (i) a higher oxidative respiration, (ii) alternative respiration and (iii) fermentation. To confirm the mRNA-seq results, a subset of twenty up-regulated transcripts were further analysed by RT-qPCR in an independent experiment at three different time points. The identification and annotation of this set of genes provides a valuable resource to understand the importance of energy sensing during mild osmotic stress.

Functional genomics studies in plants are mostly performed on model species or species characterized to a great extent. However, numerous non-model plants are important food, feed or energy sources. In addition, they may exhibit some features and processes that are unique and cannot be approached via model plants. Banana (*Musa* spp.), including the sweet and starchy types, is a typical non-model crop which ranks among the top ten staple foods, with a total production that exceeded 145 million tons in 2013 (FAOstat). Modern cultivars are hybrids from one or both major diploid ancestors, *M. acuminata* and *M. balbisiana*, which contributed the A- and B- genomes, respectively^1^. Most of these cultivars are seedless triploids (2n = 3x = 33) with an AAA, AAB or ABB genome constitution. Being highly sterile, the commercial dessert bananas are produced based on clonal propagation of only a few genotypes (Cavendish, AAA genome group). This narrow, inflexible genotypic background makes the crop more susceptible to diseases, pests and environmental issues. Therefore, the large genetic diversity in *Musa* must be exploited to move away from the few restricted commercially exploited cultivars, while still meeting consumer’s expectations.

Drought stress is one of the major abiotic factors limiting banana production. Even though the crop is grown in the humid tropics and subtropics, in many locations rainfall is not sufficient or evenly distributed throughout the year. Thus, when there is no access to irrigation, mild drought conditions are responsible for considerable yield losses. For instance, East African highland bananas (AAAh genome group) generally receive 1200–1300 mm year^−1^ and every 100 mm shortage of water induces losses of 8–10% bunch weight^2^. On the other hand, black leaf streak (better known as black Sigatoka), economically the most important fungal disease that threatens commercial banana production, thrives in humid climates whereas drier areas are natural borders for the disease. In this context, cultivating more drought tolerant bananas in drier areas with lower infection rate would become an option^3^. Hence, increasing the understanding of drought tolerance in banana at the molecular and physiological level remains a critical objective for successful, knowledge-based crop improvement and varietal selection^4^,^5^. However, the identification of drought tolerant banana varieties in natural environments remains difficult due to complications in field management, variation in phenotype and unexpected rainfall events. To facilitate the process, initial screening protocols under controlled conditions have been developed^5^ and polyethylene glycol (PEG) treatment has demonstrated to simulate the occurrence of drought stress in drying soil^6^. Genome-wide gene expression analyses in banana under abiotic stress have been sparse with only a proof a concept for drought stress with microarrays^7^ or more recent transcriptomic studies on salt or cold stress^8^,^9^, but so far no large-scale transcriptomic analysis has been reported on the response to osmotic stress.

Transcriptome research conducted in various plant species has revealed that drought stress tolerance is a multigenic trait. During the response, a large number of genes are modified in their expression involving a precise regulation of extensive gene interacting networks, which further cause a series of physiological and biochemical alterations. Initial plant response mechanisms prevent or alleviate cellular damage caused by the stress, re-establish homeostatic conditions and allow continuation of growth^10^. Therefore, equilibrium recovery of the energetic and redox imbalances imposed by the stressor are the first targets of a plant’s immediate response. Despite significant progress over the past decade aiming to understand the metabolic pathways affected by drought stress, little is known about their dynamics in non-model crops. Recent advances in next-generation sequencing (NGS) technologies and associated bioinformatic tools have revolutionized plant transcriptomics research. mRNA-seq offers a precise way to measure transcript levels while simultaneously providing sequence information^11^. This efficient, cost-effective sequencing technology has been widely used to characterize the transcriptomes of plants for gene discovery, marker development and understanding gene regulatory networks of important biological processes. However, the use of mRNA-seq to evaluate global gene expression patterns is complicated in non-model species, particularly when they are polyploid, like banana. Short reads matching multiple loci can be allocated to a single transcript or be removed from the analysis, affecting accurate quantification of expression levels. Also gene duplication and genome reorganization events contribute to such complexity^12^. The availability of a reference genome helps the alignment of reads and dealing with paralogs or allelic variants. Moreover, mRNA-seq can provide additional information to identify previously unknown or wrongly annotated coding sequences. Recently, an A- and a draft B- *Musa* genome have been released^13^,^14^, providing the first complete catalogue of all predicted genes and largely facilitating genomic/transcriptomic analyses in the genus as well as comparative studies with other plant genomes^15^.

In the present study, banana plants were exposed to 5% PEG-8000. Thus, water availability was in the mid-range of naturally occurring soil water potentials, representing mild water deficit conditions^16^. The objective of our study was to characterize the general osmotic stress reactions in banana roots. We performed large-scale transcriptome sequencing using Illumina technology on three banana genotypes representing three important subgroups of cultivated bananas with diverse genomic constitutions and different origin/geographical distribution. This work contributes to a better understanding of the molecular mechanisms and provides a workflow to study responses to water deficit in a non-model crop. We put forward that genes commonly altered in the three genotypes are more likely to play a general role in the reaction to mild osmotic stress in all banana genotypes and possibly in many crops. By selecting a subset of these genes and validating them by RT-qPCR in an independent experiment, we confirm the success of RNA-seq for transcriptome evaluation of a non-model crop.

## Results

### General landscape of the banana root transcriptome under mild osmotic stress

Using the root tip as source of mRNA, a total of 18 cDNA libraries were generated from three biological replicates of the three genotypes and the two conditions, control (0% PEG) and mild osmotic stress (5% PEG treatment). This resulted in 600 million single raw reads (100 bp) of which 94.6% passed Illumina quality filtering (Table 1). 79.6% of the high quality reads mapped to the *M. acuminata* reference genome, with about 85% of them aligning to a single location. Reads with multiple locations, ambiguous or with no match (69 million, 7%) were discarded. It reduced the number of reads that uniquely matched exons and, thus, were used for the differential expression analyses, to 383 million. On average, at least 5 reads spanned 29,931 genes, which represent 80.6% of the total number of genes in the *M. acuminata* genome (Table 1). Statistics were very similar among samples under stress and control conditions in each genotype (Supplementary Table S1).

**Figure 1:**
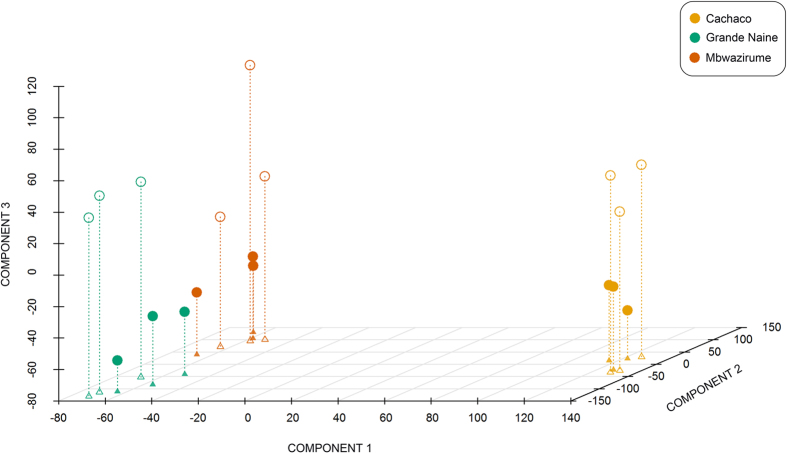
Partial Least Squares analysis of the banana root transcriptome under mild osmotic stress. Empty circles: stress conditions (5% PEG); filled circles: control conditions (0% PEG). Projections over components 1 and 2 are indicated by triangles in the 2D-space.

Results from Partial Least Square (PLS) analysis (Fig. 1) indicated that the banana root transcriptome is considerably different for A and B genomes, as component 1 noticeably separated genotype ABB (Cachaco) from both AAA genotypes (Grande Naine and Mbwazirume). Component 2 was able to distinguish between Grande Naine and Mbwazirume, which belong to different subgroups of cultivated varieties (Cavendish and East African highland bananas, respectively), as previously reported^17^. Besides, component 3 clearly separated the samples according to the treatment in all three genotypes.

### Identification, functional annotation and characterization of differentially expressed genes (DEGs)

**Figure 2:**
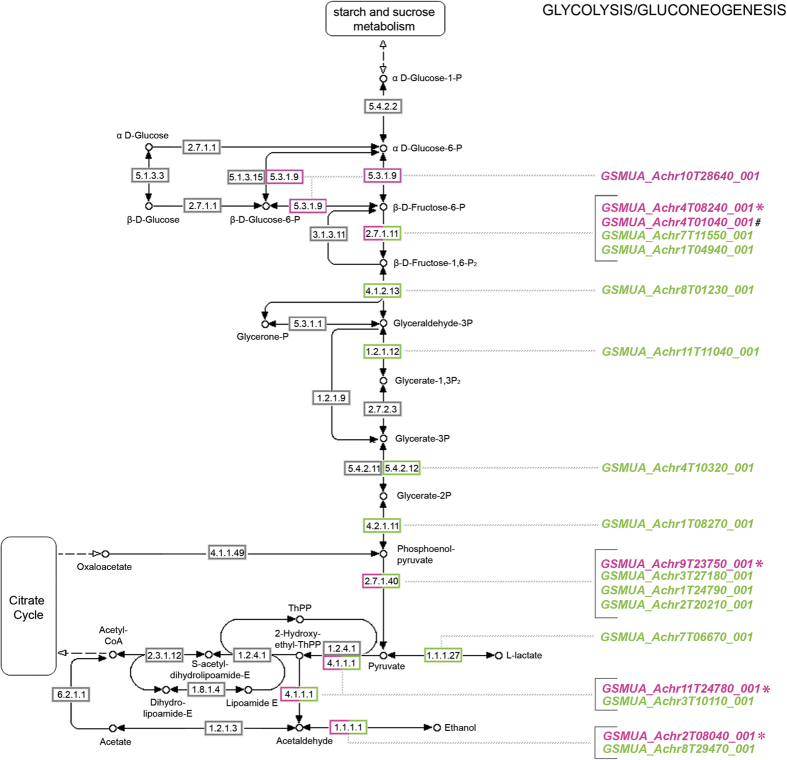
Pathway visualization of glycolysis-fermentation associated enzymes and corresponding transcripts up-regulated in banana root under mild osmotic stress (modified from KEGG pathway in plants). Enzyme codes: 5.3.1.9: Glucose-6-phosphate isomerase; 2.7.1.11: 6-phosphofructokinase; 4.1.2.13: fructose-bisphosphate aldolase; 1.2.1.12: glyceraldehyde-3-phosphate dehydrogenase; 5.4.2.12: phosphoglycerate mutase; 4.2.1.11: phosphopyruvate hydratase; 2.7.1.40: pyruvate kinase; 4.1.1.1: pyruvate decarboxylase, 1.1.1.27: L-lactate dehydrogenase; 1.1.1.1: alcohol dehydrogenase. Enzymes and transcripts coded pink are induced in all three genotypes; those coded green are induced in one or two genotypes. *genes validated by RT-qPCR. ^*#*^not detected at FDR ≤ 0.05.

As shown in Table 2, nearly double number of DEGs (670) were detected in Grande Naine as compared to Cachaco (337 DEGs) or Mbwazirume (302 DEGs). Likewise, a higher proportion of DEGs specific to Grande Naine was observed, as 563 (~84%) out of the 670 DEGs were not shared with the other two genotypes. By contrast, Cachaco and Mbwazirume showed very similar proportions (~68%) of specific DEGs, since 229 out of the 337 DEGs in Cachaco and 206 out of the 302 DEGs in Mbwazirume were distinctive of each genotype (Supplementary Figure S1). To classify the differentially expressed transcripts to putative homologs of known genes, we performed a sequence similarity search against known protein sequence datasets (NCBI nr) by using the BLASTp option from BLAST2GO^18^. In total, 319 (94.7%) of the differentially expressed transcripts in Cachaco, 636 (94.9%) in Grande Naine and 292 (96.7%) in Mbwazirume showed significant (e-value < 10^−3^) sequence similarity to entries of NCBI nr (Supplementary Table S2). Our aim is to characterize the differential transcriptome for banana in general so we focus further on the DEGs commonly detected in all three genotypes. Using the union of the results provided by two different statistical approaches (see Methods section), 92 DEGs were detected in all three genotypes (Supplementary Figure S2). BLASTp results from BLAST2GO identified homologous proteins for 89 (96.7%) of them and gene ontology (GO) terms were assigned to 81 (91%) out of the 89 genes. Additionally, enzyme codes were assigned to 26 (32.1%) out of the 81 genes with associated GO terms (Supplementary Table S3). GO enrichment analysis was performed to discover significantly over-represented functional categories by comparing the annotated set of DEGs to all banana genes (GO terms were available for 26,097 of the 36,542 sequenced genes). In total, 24 GO terms were significantly enriched using Fisher’s exact test at *p* < 0.01 (Table 3). Overrepresented GO terms in the mRNA-seq data provides new insights into mild osmotic stress-induced processes and functions. Most significant enriched GO terms related to biological processes were grouped into “response to (low) oxygen levels” (GO:0001666, *p*-value: 5.0 × 10^−5^; GO:0036293, *p*-value: 5.3 × 10^−5^ and GO:0070482, *p*-value: 5.9 × 10^−5^), “oxidation-reduction process” (GO:0055114, *p*-value: 2.5 × 10^−4^), “protein hydroxylation” (GO:0018126 and GO:0019511, *p*-value: 5.2 × 10^−4^) and “metabolic processes” (GO:0044710, *p*-value: 5.2 × 10^−4^ and GO:0006091, *p*-value: 1.3 × 10^−3^). This enrichment analysis points towards an important function for classes associated with respiration, glycolysis and fermentation. KEGG pathway analyses using the DEGs revealed that glycolysis coupled to fermentation was significantly induced after three days of 5% PEG treatment (Fig. 2; Supplementary Table S4). To gain a broader overview of the changes in the pathway, all *Musa* genes coding for glycolytic and fermentative enzymes were checked in each genotype. Six genes corresponding to five different enzymes were induced in the three genotypes. Additionally, twelve genes corresponding to nine enzymes showed up-regulation in one or two genotypes. In total, eighteen genes corresponding to ten important enzymatic steps in the glycolysis-fermentation pathway were up-regulated in at least one genotype (Fig. 2). In the steps catalysed by 6-phosphofructokinase (EC 2.7.1.11), pyruvate kinase (EC 2.7.1.40), pyruvate decarboxylase (EC 4.1.1.1) and alcohol dehydrogenase (EC 1.1.1.1), more than one gene encoding for the same enzyme showed increased expression.

### Validation of up-regulated genes by quantitative real time RT-PCR (RT-qPCR)

**Figure 3:**
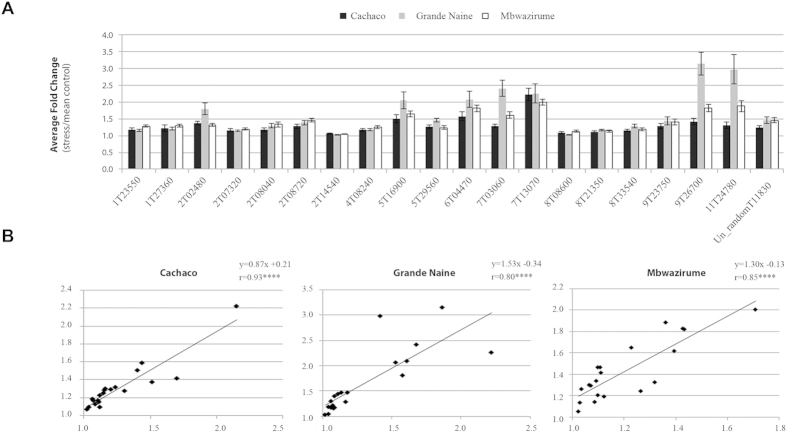
Confirmation of expression profiles by RT-qPCR for the 20 selected candidate genes and comparison with RNA-seq data. **(A)** Relative expression levels (fold changes of log transformed data) at day 3; gene ID abbreviations according to Table 4. *Musa* genes *EF-1*, *L2* and *ACT-1* were used as internal controls to normalize the expression data. (**B)** Correlations between RNA-seq and RT-qPCR results at day 3 in each genotype. X-axis: average fold change (stress vs. mean control) in RNA-seq; Y-axis: average fold change (stress vs. mean control) in RT-qPCR; error bars: standard error of the means in each genotype; r: Pearson correlation coefficient; ****p < 0.0001.

To confirm the accuracy and reproducibility of the mRNA-seq results and the robustness of the statistics, a subset of twenty genes commonly up-regulated in the three genotypes (Table 4) was selected as described in Methods section for validation by RT-qPCR. Therefore, an independent experiment was set up with 6 biological replicates per genotype and three different time points: 6 hours, 3 days and 7 days after 0 and 5% PEG treatment. ANOVA test on the RT-qPCR data indicated a significant genotype-independent treatment effect (*p* < 0.05) for 18 genes at the earliest time point (6 h) and for all 20 genes at day 3 and day 7 (Table 5, Supplementary Table S5 and Supplementary Figure S3). At day 3 (same time point as the mRNA-seq results), treatment effects were significant (*p* < 0.05) in all three genotypes for 18 out of the 20 candidate genes (Supplementary Figure S3). In general, fold changes of RT-qPCR expression values were very similar among the three genotypes (Fig. 3A). Besides, highly significant correlation coefficients were found (r = 0.80–0.93; *p* < 0.0001) when comparing RNA-seq and RT-qPCR results in each genotype, indicating a good consistency between the two analysis techniques and the two independent experiments (Fig. 3B). Similar correlation coefficients between mRNA-seq and RT-qPCR have been obtained in recent transcriptomic studies conducted in other plant crops^19^,^20^.

### Identification of *Musa* paralogs and corresponding gene expression patterns

To infer as accurately as possible the functions of the candidate genes, all genes with similar sequences were identified in the *Musa* genome and their orthology relationships established with genes from *Arabidopsis thaliana*. For each candidate, one or more paralogs (i.e. genes derived by duplication in the *Musa* specific lineage) were identified (Table 4). As shown for the genes related to the glycolysis-fermentation pathway, paralogs can exhibit an expression pattern significantly correlated (*p *< 0.01) to that of the candidate gene or can show different expression patterns (Supplementary Figure S4). In the first case, the genes appear as redundant copies (at least in the tissues and conditions analysed) whereas sub-functionalization after duplication events can be postulated for the genes whose expression pattern diverged^21^.

## Discussion

### Transcriptome analysis in *Musa* under of mild osmotic stress

In this study, mRNA-seq was used to analyse transcriptomic changes in the roos of three triploid banana genotypes subjected to mild osmotic stress. The biggest challenge to perform mRNA-seq on a non-model crop, such as banana, is its ploidy level. A few studies of the banana transcriptomic response to abiotic/biotic stresses have been reported for triploid genotypes^9^,^22^,^23^ and different approaches were applied depending on the availability or not of the diploid reference genome. Here we opted for a mapping-first approach of short reads^12^. As expected, the percentage of reads that mapped to the reference genome was higher for both AAA genotypes (84%) than for ABB Cachaco (70%), since the latter contains two copies of the B genome and only one copy of the A genome (Table 1). However, the number of high quality reads and mapped genes was comparable among the three genotypes and ensured a good coverage of the *Musa* genome (Table 1 and Supplementary Table S1).

The varying number of DEGs in the three genotypes and the Partial Least Square analysis (Fig. 1) point towards genotype specific reactions. Provided similar percentage of mapped reads in Grande Naine and Mbwazirume (both AAA, Table 1), Grande Naine seems to be the most reactive genotype, since nearly double number of DEGs were detected when comparing to Cachaco or Mbwazirume (Table 2).

### Physiological impact of mild osmotic stress

#### Enhanced oxidative respiration and reactive oxygen species (ROS) production

Stress responses in plants occur at various organ levels, among which root specific processes are particularly relevant^24^. Roots are big sinks of energy and the main consumers of carbon fixed in photosynthesis during the vegetative stage. During stress, a higher proportion of dry matter is allocated to the root in order to satisfy its increased energy demand^25^. As non-green tissues, roots entirely depend on glycolysis and mitochondrial respiration for their energy production. Stress causes a higher energy consumption and, thus, enhances respiration, one of the major cellular pathways dependent on oxygen^26^. Three *Musa* genes identified in our study (*GSMUA_Achr9T26700_001*, *GSMUA_Achr5T16900_001* and *GSMUA_Achr5T29560_*001) are involved in mitochondrial respiration (Table 4).

**Figure 4:**
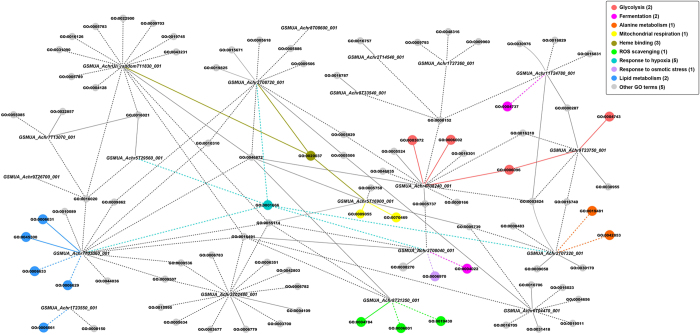
Interactive network of candidate genes and associated GO terms depicting key root processes affected under mild osmotic stress. Solid lines: GO terms assigned via Uniprot (http://www.uniprot.org/). Dashed lines: GO terms assigned via cross-species annotation. Relevant GO terms are highlighted in different colors and the number of genes associated to them is indicated between brackets.

Gene *GSMUA_Achr9T26700_001*, (Fig. 4), shows a strong induction in all three genotypes (Table 4 and Supplementary Figure S3). It has been annotated as a subunit of the respiratory complex I, also known as NADH:ubiquinone oxidoreductase (Table 4 and Supplementary Table S3), a major component of the mitochondrial electron transport chain. This complex couples the oxidation of NADH to the reduction of ubiquinone with the generation of a proton gradient used for ATP synthesis^27^. We found one paralogous gene, *GSMUA_Achr6T27380_001*, that is also strongly up-regulated in the three genotypes and shows an expression pattern significantly correlated (*p * < 0.01) to that of *GSMUA_Achr9T26700_001* (Supplementary Figure S5).

Gene *GSMUA_Achr5T16900_001*, associated to electron carrier activity and the respiratory chain (GO:0009055 and GO:0070469; Fig. 4), codes for a cytochrome *c* (Table 4 and Supplementary Table S3), which is involved in electron transfer between the respiratory complex III (ubiquinone-cytochrome *c* oxidoreductase) and complex IV (cytochrome *c* oxidase (COX))^28^.

Gene *GSMUA_Achr5T29560_001*, related to integral component of membrane (GO:0016021; Fig. 4), has been annotated as an hypoxia responsive family gene (Table 4) and as a respiratory super complex factor 2 (Rcf2) homolog (Supplementary Table S3). Rcf2 is a cytochrome *c* oxidase (COX) subunit required for optimal enzyme activity and the correct assembly of the cytochrome bc1-COX super complex, which belongs to the conserved hypoxia-induced gene 1 (Hig1) protein family^29^. We have found other three *Musa* paralogs with a significantly correlated (*p *< 0.01) expression pattern to that of *GSMUA_Achr5T29560_001* (Supplementary Figure S6).

The identification of *GSMUA_Achr9T26700_001*, *GSMUA_Achr5T16900_001* and *GSMUA_Achr5T29560_001* and of some paralogs with correlated expression patterns supports the assumption of an enhanced respiration rate under mild osmotic stress. We hypothesize that the enhanced respiration is driven by the increased energy demand and serves to cope with the adverse conditions^25^.

In stressed plants, a direct link between the mitochondrial electron transport respiratory chain and ROS production has been demonstrated. ROS can act as important signalling molecules involved in the stress signal transduction pathway, while excessive ROS may induce oxidative damage to cellular components and structures^30^. Plants have developed an antioxidant system to remove the excess of superoxide (O_2_^−^) radicals, a type of ROS, which includes superoxide dismutases. These enzymes convert toxic O_2_^−^ to hydrogen peroxide and water^31^. Gene *GSMUA_Achr8T21350_001* was annotated to superoxide dismutase activity (GO:0004784; Fig. 4) and, thus, to ROS scavenging, and described as a superoxide dismutase. The gene shows a significantly induced expression in each genotype after 3 days of PEG treatment and in two genotypes after 7 days of PEG treatment, while no significant induction was detected in the earliest time point (Supplementary Figure S3). The product of *AT3G10920*, ortholog in *Arabidopsis* (Table 4), is a manganese superoxide dismutase (MSD) located in the mitochondria which also accumulates under osmotic stress^31^. We suggest that banana roots trigger the complex antioxidant network to regulate ROS production and to facilitate appropriate signalling during mild osmotic stress.

#### Fermentation and carbon allocation

Metabolically active cells, such as those in the root tip, have a high oxygen demand and are particularly prone to suffer from hypoxia, i.e. low oxygen levels^32^. According to Aguilar *et al.* (2003), respiratory oxygen consumption in banana roots decreases substantially with distance from the apex and the stele^33^. We hypothesize that higher respiration rates in both, the root apex and the stele, lead to a shift from more aerobic to more anaerobic metabolism for ATP production. The options are alternative respiration and NAD^+^ regeneration via fermentation^34^. Fermentative ATP production is much less efficient and increases the demand for carbohydrates. Consistently, we observed a generalized induction of transcripts for enzymes involved in glycolysis and fermentation (Fig. 2 and Supplementary Table S4). Protein accumulation of enzymes belonging to this pathway has also been reported in banana plants and meristems under osmotic stress^5^,^35^ and, recently, an increase of glycolysis-related proteins has been found in soybean roots submitted to drought stress^36^. In our study, there were multiple steps in the glycolysis-fermentation pathway where genes were significantly induced (Fig. 2). The increased expression of the genes *GSMUA_Achr4T08240_001* (6-phosphofructokinase; 6PFK), *GSMUA_Achr9T23750_001* (pyruvate kinase; PK), *GSMUA_Achr11T24780_001* (pyruvate decarboxylase; PDC) and *GSMUA_Achr2T08040_001* (alcohol dehydrogenase; ADH) has been verified by RT-qPCR (Supplementary Figure S3). All 4 genes were annotated to the glycolytic process (GO:0006096) and *GSMUA_Achr11T24780_001* (pyruvate decarboxylase; PDC) and *GSMUA_Achr2T08040_001* (alcohol dehydrogenase; ADH) are involved in the ethanolic fermentation process (Fig. 4). Since 6-phosphofructokinase, pyruvate kinase and pyruvate decarboxylase catalyse irreversible reactions, they represent important control points. The up-regulation of the genes under stress ensures a continuous flow of metabolites throughout the pathway. This is supported by the fact that all four candidate genes have at least another paralog with a significantly correlated (*p *< 0.01) expression pattern (Supplementary Figure S4). Pyruvate, the final product of glycolysis, can either be converted into lactate by lactate dehydrogenase or to ethanol by pyruvate decarboxylase and alcohol dehydrogenase. As an initial reaction to oxygen deprivation, lactic acid fermentation is activated causing a decrease in cytosolic pH. This reduces the activity of the responsible enzyme and lactic acid fermentation is followed by alcoholic fermentation^37^. The observed up-regulation of genes encoding pyruvate decarboxylase and alcohol dehydrogenase in *Musa* is in agreement with previous studies where the corresponding *Arabidopsis* orthologs also showed induction under low oxygen conditions^38^,^39^. Interestingly, ethanol production and alcohol dehydrogenase induction was also found in plants under other abiotic stresses, including dehydration^40^,^41^, which confirms the hypothesis that fermentation plays a role under environmental stress. In our study, the majority of up-regulated genes involved in glycolysis were predicted to be in the cytosol (Supplementary Table S4), an indication that most of the carbon source is channeled via cytosolic glycolysis to feed fermentative pathways. Plants regulate the balance between respiration and fermentation to be able to control the internal oxygen level^42^. The induction of the fermentative enzymes pyruvate decarboxylase and alcohol dehydrogenase is not exclusively dependent on the oxygen concentration, but is also linked to changes in the energy status (ratio of ATP to ADP). Consequently, sensing the energy status would be an important component for optimizing plant metabolism.

Pyruvate can also serve as precursor for the synthesis of alanine by the enzyme alanine amino transferase (AlaAT). This enables to conserve carbon skeletons that otherwise would be lost by fermentation and also prevents cytoplasmic acidification by avoiding lactic acid production and proton consumption^43^,^44^. Other possible benefits of alanine accumulation during hypoxia have been reviewed by Menegus *et al.* (1993) and include prevention of ammonium toxicity, provision of a reduced nitrogen store and generation of osmotic pressure^45^. Hypoxia induced transcripts of genes encoding alanine amino transferases has been reported in other plant species as *Arabidopsis*, wheat or soybean^39^,^46^,^47^. Gene *GSMUA_Achr2T07320_001* has been annotated as alanine amino transferase (AlaAT; Fig. 4, Table 4 and Supplementary Table S3). The *Arabidopsis* ortholog *AT1G17290* also shows an induction under low oxygen conditions^38^,^39^ and, consistently, we observed up-regulation of *GSMUA_Achr2T07320_001* already at 6 hours of PEG treatment (Supplementary Figure S3). This suggests that the banana root tip starts rebalancing carbon allocation as soon as 6 hours. 

#### Alternative respiration and haemoglobin (Hb)/nitric oxide (NO) cycle

As an alternative to oxygen-based respiration and classic fermentation, a process involving stress-induced class I haemoglobins (Hbs) has been described in plants under low oxygen^48^. Under such conditions, root mitochondria use nitrite as final electron acceptor instead of oxygen producing NO^49^, which is toxic to cells and scavenged by haemoglobin proteins. In *Arabidopsis* and barley plants under hypoxia, a rapid induction of haemoglobin expression has been detected^50^,^51^. However, this induction would rather respond to cell energy/redox status than to low oxygen levels^52^. Interestingly, overexpression of haemoglobin in hypoxic maize cell cultures resulted in lower ethanolic fermentation, since a greater turnover of NO in the Hb/NO cycle increased NADH oxidation, replacing to some extent the requirement for alcohol dehydrogenase activity^53^. Gene *GSMUA_Achr2T08720_001*, connected to oxygen binding/transport and heme binding (GO:0019825, GO:0015671 and GO:0020037; Fig. 4), has been annotated as class I nonsymbiotic haemoglobin (Table 4 and Supplementary Table S3) and its *Arabidopsis* ortholog *AT2G16060* is also up-regulated under hypoxia^50^. Remarkably, cytochrome b5 reductases have been proposed to play a role in the NO/Hb cycle, particularly in the reduction of metahaemoglobin to haemoglobin^48^. According to this hypothesis, up-regulation of *GSMUA_AchrUn_randomT11830_001*, also connected to heme binding (GO:0020037; Fig. 4) and annotated as a member of the cytochrome b5 (Table 4 and Supplementary Table S3), could be linked to the induction of our haemoglobin gene (*GSMUA_Achr2T08720_0010*).

#### Detoxification

Apart from ROS and NO, other compounds can be toxic to cells when they accumulate in the mitochondria. An example is hydrogen sulfide, generated from cysteine degradation and considered a potent inhibitor of aerobic respiration. Its effects change from physiological to toxic within a narrow concentration range. Thus, regulatory mechanisms are needed to keep endogenous sulfide levels under control^54^. Gene *GSMUA_Achr1T27360_001* has been annotated as *ETHE1*-like, a member of the b-lactamase fold superfamily (Table 4 and Supplementary Table S3). The ortholog in *Arabidopsis* is *AT1G53580*, which encodes a mitochondrial sulfur dioxygenase (*AtETHE1*) involved in detoxification of hydrogen sulfide^55^. *AtETHE1* and *OsETHE1* from rice have proved to show high root-specific and stress-inducible expression, suggesting a potential role of this gene in the stress response^56^,^57^. Due to its higher metabolism, the root tip undergoes quick protein turnover and sulfate acquired by roots constitute the primary sulfur source for growth, justifying the role of *ETHE1* as part of the sulfur catabolism pathway in roots. Recently, a key function in the use of amino acids as alternative respiratory substrates during carbohydrate starvation has been attributed to *AtETHE1*^58^.

#### Other root transcripts up-regulated during mild osmotic stress

One of our candidates, *GSMUA_Achr6T04470_001*, was linked to oxidoreductase activity (GO:0016706; Fig. 4) and has been annotated as a prolyl-4-hydroxylase alpha subunit (P4H; Table 4 and Supplementary Table S3). In a study carried out in chickpea roots after dehydration treatment, prolyl 4-hydroxylase alpha subunits belonged to the most up-regulated group of transcripts^59^. Interestingly, overexpression in *Arabidopsis* of different *AtP4H* genes has proven to increase root hair length/density, a response that would facilitate both nutrient and water assimilation by the plant^60^. Gene *GSMUA_Achr2T14540_001*, associated to the transfer of glycosyl groups (GO:0016757; Fig. 4), has been annotated as a glycosyl transferase 61 family protein (Table 4 and Supplementary Table S3). Differential expression of glycosyl transferases was also found in *Arabidopsis* root cultures under hypoxia treatment and in cotton roots subjected to drought stress, where they were linked to cell wall processes^38^,^61^. Gene *GSMUA_Achr7T03060_001*, related to fatty acid metabolism and desaturase activity (GO:0006631 and GO:0045300; Fig. 4), has been annotated as a stearoyl-[acyl-carrier-protein] 9-desaturase (Table 4 and Supplementary Table S3), a gene involved in the biosynthesis of polyunsaturated fatty acids^62^. Its paralog, *GSMUA_Achr8T32640_001*, is also up-regulated under the applied stress and exhibits an expression pattern significantly correlated (*p *< 0.01) to that of *GSMUA_Achr7T03060_001* (Supplementary Figure S7). Recently, the ortholog in *Arabidopsis*, *AT1G43800*, has been shown to increase the levels of unsaturated fatty acids in crown galls under hypoxia and drought stress conditions^63^. Remarkably, candidate *GSMUA_Achr1T23550_001* (gene of unknown function, Table 4) has also been associated to lipid metabolism (GO:0006661; Fig. 4). Gene *GSMUA_Achr7T13070_00* is linked to transmembrane transporter activity and integral component of membrane (GO:0022857 and GO:0016021; Fig. 4), and has been annotated as a nodulin MtN21-like transporter family protein with a strong induction in the three analysed genotypes (Table 4). Interestingly, *AT1G75500*, another nodulin MtN21-like protein, has been found up-regulated in *Arabidopsis* root cultures under hypoxia, but its specific involvement in the stress response has not yet been determined^38^.

Given the advances in genomic technology platforms, the unique ability to compare transcriptomes across several species can be exploited to cross-reference information concerning genes and gene functions. However, it is still a challenge to infer gene functions in a non-model crop and to rely in cross-species annotation, as exemplified for candidates *GSMUA_Achr1T23550_001* (GO:0006661 and GO:0008150; Fig. 4) and *GSMUA_Achr8T08600_001* (GO: 0005886; Fig. 4), since their gene functions could not be inferred even when comparing with *Arabidopsis* or other plant species (Table 4 and Supplementary Table S3).

## Conclusion

**Figure 5:**
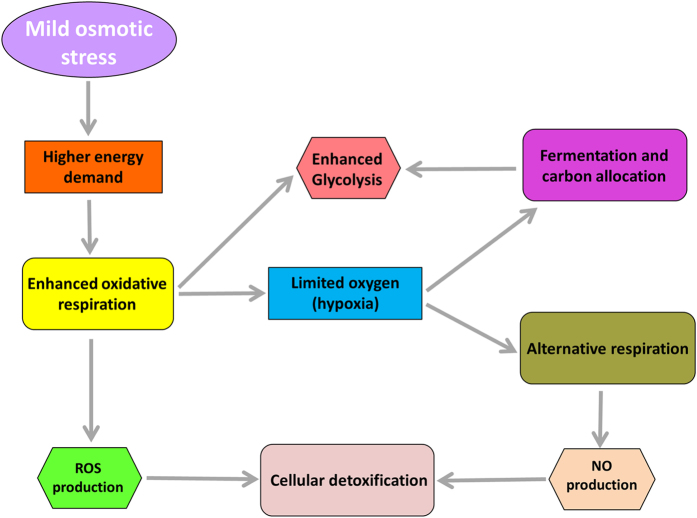
Summary of the main processes induced during osmotic stress in banana roots. Mild osmotic stress causes a higher energy demand which enhances aerobic respiration and leads to local hypoxia in the root tip. In this situation, alternative respiration and fermentation/carbon allocation take place. Due to the induction of fermentation and the increase in respiration, the glycolytic pathway is also enhanced. Respiratory activity generates toxic compounds, as reactive oxygen species (ROS) and nitric oxide (NO), which are detoxified by cells.

Transcriptome profiling in the polyploid non-model crop *Musa* indicated that the roots change the broad spectrum of energy metabolism after applying mild osmotic stress. We hypothesize that osmotic stress leads to a drop in energy level, which induces a metabolic shift towards (i) a higher oxidative respiration, (ii) alternative respiration and (iii) fermentation (Fig. 5). By validating a subset of genes by RT-qPCR, we confirm the success of RNA-seq for evaluation of a non-model crop. This work contributes to a better understanding of the molecular mechanisms and provides a workflow to study responses to osmotic stress.

## Methods

### Plant material, growth conditions and osmotic stress treatment

*In vitro* banana plants of the genotypes ‘Cachaco’ (Bluggoe ABB, ITC0643), ‘Grande Naine’ (Cavendish AAA, ITC0180) and ‘Mbwazirume’ (East African highland banana AAAh, ITC0084) were supplied by the Bioversity International Transit Centre hosted at KU Leuven, Belgium. *In vitro* plants were grown for 35 days in autotrophic conditions prior to the start of the experiment and roots were covered to protect from light. Each plant (3 biological replicates per genotype) was grown in a 500 mL PP container with 305 mL medium: 3.61 g/L KNO_3_, 1.21 g/L K_2_SO_4_, 1.61 g/L MgSO_4_. 7H_2_O, 1.81 g/L MgCl_2_.6H_2_O, 0.6 g/L Sequestrene, 0.0114 g/L H_3_BO_3_, 0.027 g/L MnSO_4_.H_2_O, 0.0023 g/L ZnSO_4_.7H_2_O, 0.0016 g/L CuSO_4_.5H_2_O, 0.0007 g/L NaMo_4_.2H_2_O, pH = 6 (modified from Swennen *et al.*^64^). Plants were grown in a phytotron (Aralab Fitoclima Bio 600) with a 12 h/12 h light/dark period (average light intensity of 183 ± 29 μmol photons m^−2 ^s^−1^). Humidity and temperature were kept constant at 75% and 25 °C, respectively. At the start of the treatment, the group of the stressed plants received fresh medium containing five percent (W/W) of PEG-8000 (Sigma, USA), while control plants received the same fresh medium without PEG-8000. Subsequently, in both groups (stress/control), the medium was refreshed when it reached 55% of the initial volume in at least one plant. Three days after 5% PEG treatment the plants were sacrificed and root tips (segments of on average 4 cm from the apex) were collected for RNA-sequencing. An independent experiment was set up for RT-qPCR validation, including six biological replicates per genotype and three different time points: 6 hours, 3 days and 7 days after 5% PEG or control treatment.

### RNA extraction and cDNA library preparation

Root material from the two independent experiments was snap frozen in liquid nitrogen and stored at −80 °C. Total RNA was extracted as described in^65^. Samples were treated with TurboTM DNase I (Ambion, Austin, TX, USA) for 45 min at 37 °C followed by a phenol-chloroform/ethanol purification step to eliminate gDNA traces. Real-time PCR was performed using DNase-treated RNA as template and primers for the Elongation factor 1α (*EF1*) genomic sequence to verify absence of gDNA. Only samples with undetected amplification after 40 cycles were used. RNA quantity and quality (A260/230, A260/280) were determined using Nanodrop ND-1000TM spectrophotometer (Thermo Fisher Scientific, Wilmington, DE, USA).

For cDNA library construction, RNA integrity was checked by ExperionTM (BIO-RAD Laboratories, Inc. USA; RQI > 9.4) and BioAnalyzer (Agilent;RIN > 7.8). The TruSeq RNA Sample Seq kit (Illumina Inc.) was used according to the manufacturer’s protocol to generate the libraries. In brief, poly-A containing RNA molecules were purified from 1 μg total RNA using oligo-dT magnetic beads and fragmented by adding the fragmentation buffer and heating at 94 °C for 8 min in a thermocycler. First strand cDNA was synthesized using random primers. Second strand cDNA synthesis, end repair, A-tailing and adapter ligation was done in accordance with the manufacturer’s instructions. Purified cDNA templates were enriched by 15 cycles of PCR for 10 s at 98 °C, 30 s at 65 °C and 30 s at 72 °C using Illumina’s proprietary primers and Phusion DNA polymerase. Each indexed cDNA library was verified using a DNA 1000 Chip on a Bioanalyzer 2100, quantified by RT-PCR with the KAPA Library Quantification Kit for Illumina Sequencing Platforms (Kapa Biosystems Ltd, SA) and diluted to 10 nM using Illumina’s resuspension buffer.

### Illumina sequencing and mapping

Multiplex sequencing on an Illumina HiSeq2000 was performed as 100 bp, single reads at GenomiX, Montpellier, France. For each lane of sequencing, 5 libraries were equimolarly pooled, denatured using 0.1N NaOH and diluted to a final concentration of 7 pM using Illumina’s HT1 buffer. 120 μL of the dilution was then transferred into a 200 μL strip tube and placed on ice before loading onto the cBot. The flow cell was clustered using Single Read Cluster Generation Kit (Illumina Inc.), according to the Illumina SR_amplification_Linearization_Blocking _PrimerHyb recipe. The flow cell was loaded onto the Illumina HiSeq 2000 instrument following the manufacturer’s instructions and sequencing was performed with the 100 cycles, single read, indexed protocol. Image analyses and base calling were performed using the HiSeq Control Software (HCS) and Real-Time Analysis component (RTA). Demultiplexing was carried out using CASAVA. Data quality was assessed using fastQC (Babraham Institute, USA) and the Illumina software SAV. RNA-seq reads were quality filtered using Illumina purity filter and aligned to the *M. acuminata* assembly v1^13^ from the Banana Genome Hub^66^ with gene model annotations, using the splice junction mapper TopHat 1.4.1^67^ and Bowtie 0.12.8 (default parameters). Gene counting was calculated using HTSeq v0.5.3p9 (http://www-huber.embl.de/users/anders/HTSeq/) in union mode. Reads not aligned on exons or with multiple hits were disregarded.

### Differential gene expression analyses and selection of candidate genes

Differential gene expression between stress and control conditions was evaluated using edge R v2.6.2^68^ in the R statistical environment v2.15.0^69^, and data were normalized using RLE^70^. *p*-value was adjusted for multiple testing by controlling the false discovery rate (FDR) at ≤5%. A second method was applied to detect DEGs. For the normalization, raw read counts were divided by the total number of reads in each library and results were log-transformed to assess normality by Shapiro-Wilk test^71^ using STATISTICA 7.0 (StatSoft, Inc. USA). As normality was not always achieved and due to the limited number of biological replicates, the non-parametric Kolmogorov-Smirnov test^72^ was applied (*p *< 0.1) using STATISTICA 7.0 (StatSoft, Inc. USA). Partial Least Square Analysis (PLS) was carried out to reveal the most important variables (genes) and to provide a rank to all DEGs^73^. A total of 20 candidate genes up-regulated in the three genotypes were selected for subsequent analyses. Of them, 4 were detected by edgeR-RLE, 6 by the non-parametric test and Partial Least Square Analysis, and 10 by both methods simultaneously. Differential expression analyses and Partial Least Square Analysis were performed excluding genes with less than 15 reads in at least one genotype when combining stress and control libraries. In total 7,680 transcripts (20.4% of all sequenced transcripts) were excluded.

### Identification of orthologous and paralogous genes

For each candidate gene, a genome wide analysis was performed to identify putative *Musa* paralogs, according to first clustering level in GreenPhyl DB^74^. Annotations were manually inspected and, when necessary, corrections were made based on mapping data of the RNA-Seq libraries and comparisons with similar genes annotated in *Vitis vinifera* and *A. thaliana.* Following an approach based on definition of multi-specific orthologous groups^15^, for each candidate the orthologous gene(s) was identified in *A. thaliana*. All *Musa* genes included in the same orthologous group were considered real paralogs. To identify significant correlations between expression levels of the candidate genes and their corresponding paralog(s), Spearman rank correlation analysis (*p *< 0.01) on the normalized read counts were performed in R package.

### Annotation, GO enrichment and pathway analysis

For the DEGs, protein homologues were searched using NCBI Blast option from BLAST2GO^18^, which examined sequences against public non-redundant databases using BLASTp algorithms (e-value < 10^−3^). Gene ontology (GO) terms were assigned and clustered based on biological process, cellular component or molecular function. Mapping and annotation were performed using default parameters (e-value hit filter 10^−6^, annotation cutoff 55, GO weight 5, HSP-hit-coverage cut-off 0). Alternatively, GO terms for the selected candidate genes were retrieved from Uniprot^75^. Specific gene products were associated to biological pathways as determined by the KEGG pathway mapping function^76^ from BLAST2GO. For the candidate genes, associated GO terms obtained via Uniprot and/or BLAST2GO were imported into Cytoscape to generate the corresponding interaction network^77^. GO term enrichment analysis was conducted using the R package TopGO^78^ and significance was calculated based on Fisher’s exact test with a cut-off threshold of *p* < 0.01.

### Design and optimization of RT-qPCR primers

For each candidate gene and the *Musa* reference genes ribosomal protein L2 (*L2*), Actin-1 (*ACT-1*), Tubulin β-1 chain (*TUB1*) and Elongation factor 1α (*EF1*), copy-specific primers were designed to amplify part of the 3′ or 5′ untranslated regions using Primer3 (version 0.4.0). Chosen parameters were: product size range 100–150 bp, primer size 20–22 bp, primer Tm 57–60 °C (with maximum Tm difference = 2 °C) and GC content 45–60%. Primer combinations were custom-ordered from a commercial supplier (Integrated DNA Technology, USA) and tested at two concentrations (100 and 150 nM) and with two cDNA dilutions (x3 and x48). Amplicon sizes were checked by 2% agarose gel electrophoresis and ethidium bromide staining. Primer specificities were confirmed with the melting-curve after amplification by RT-qPCR. A standard curve of five serial four-fold dilutions of pooled cDNA, a no template control, was made to calculate gene-specific amplification efficiencies (E) and correlation coefficients (R^2^) (Supplementary Table S6).

### RT-qPCR analysis and determination of gene expression levels

For RT-qPCR validation of RNA-seq data, root tip RNA was isolated from the independent experiment. For each DNA-free RNA sample, 1 μg was reversed-transcribed to cDNA by using an oligo(dT)18 primer and the RevertAid H Minus First Strand cDNA Synthesis kit (Fermentas, St-Leon Rot, Germany) according to the manufacturer’s instructions. RT-qPCR design, calculations and statistics used followed the MIQE guidelines^79^. RT-qPCR was carried out in 96-well plates and in duplicated volumes of 15 μL using the SYBR Green I technology. All reactions were analysed with the StepOnePlusTM Real-Time PCR System (Applied Biosystems, USA). The master mix containing 1 × ABsoluteTM QPCR SYBR® Green Mix (Thermo Scientific, Epsom, UK), 100 or 150 nM of each forward and reverse primer (Supplementary Table S6) and 125 ng λ-DNA (Roche Diagnostics, Vilvoorde, Belgium) was mixed with 2 μL of a 50x diluted template cDNA, control gDNA or water. λ-DNA was added as carrier DNA to minimise absorption and Poisson effects. The following amplification program was used: polymerase activation at 95 °C for 15 min., 40 cycles of 95 °C for 15 s, 60–62 °C for 20 s and 72 °C for 20 s. A standard curve as the above mentioned and the cDNA samples were run concomitantly in each assay. Cq values were imported into qBase+ software (Biogazelle) and relative expression values were determined using the most stable reference genes (*EF-1*, *L2* and *ACT-1*). Data were log-transformed to assess normality by Shapiro-Wilk test using STATISTICA 7.0 (StatSoft, Inc. USA). As normality was achieved, a two-way analysis of variance (ANOVA; *p *< 0.05) was applied to calculate the effects of the genotype, treatment and genotype × treatment using STATISTICA 7.0 (StatSoft, Inc. USA). Fisher’s least significant difference (LSD) mean comparison was used as the post-hoc test.

## Additional Information

**How to cite this article**: Zorrilla-Fontanesi, Y. *et al.* Differential root transcriptomics in a polyploid non-model crop: the importance of respiration during osmotic stress. *Sci. Rep.* **6**, 22583; doi: 10.1038/srep22583 (2016).

## Supplementary Material

Supplementary figures

Supplementary tables

## Figures and Tables

**Table 1:** Summary of the banana transcriptome sequencing dataset.

Genotype	aCachaco (ITC0643)	aGrande Naine (ITC0180)	aMbwazirume (ITC0084)	bTotal/cAverage
No. raw reads (10^6^)	194	214	192	600^b^
No. high-quality reads (10^6^) (and %)	185 (95)	201 (94)	182 (95)	568 (94.6)^b^
No. aligned reads (10^6^) (and %)	130 (70)	169 (84)	153 (84)	452 (79.6)^b^
No. unique reads on exons (10^6^)	108	143	132	383^b^
Genes covered (%)	79	81.8	81	80.6^c^
Average No. reads by transcript	486	640	593	573^c^
Median No. reads by transcript	106	140	128	125^c^

^a^Average values of the 6 libraries per genotype.

^b^Total or.

^c^average values of the three genotypes.

**Table 2:** Number of differentially expressed, up- and down-regulated genes detected by edgeR-RLE in the root of each genotype during mild osmotic stress.

Genotype	No. DEGs	No. Up-regulated genes	No. Down-regulated genes
Cachaco (ABB)	337	260	77
Grande Naine (AAA)	670	310	360
Mbwazirume (AAA)	302	251	51

Number of biological replicates (stress/control): n = 3/3; False discovery rate (FDR) ≤ 0.05; DEGs: differentially expressed genes.

**Table 3:** Enriched GO terms (over representation) for biological processes detected by Fisher’s Exact Test at p < 0.01 on TopGO package^78^.

GO ID	Term	Annotated^a^	Significant^b^	Expected^c^	Significance
GO:0001666	response to hypoxia	71	4	0.2	5.00 × 10^−5^
GO:0036293	response to decreased oxygen levels	72	4	0.21	5.30 × 10^−5^
GO:0070482	response to oxygen levels	74	4	0.21	5.90 × 10^−5^
GO:0055114	oxidation-reduction process	1922	15	5.47	2.50 × 10^−4^
GO:0018126	protein hydroxylation	12	2	0.03	5.20 × 10^−4^
GO:0019511	peptidyl-proline hydroxylation	12	2	0.03	5.20 × 10^−4^
GO:0044710	single-organism metabolic process	6929	32	19.73	9.40 × 10^−4^
GO:0006091	generation of precursor metabolites and energy	759	8	2.16	1.35 × 10^−3^
GO:0044699	single-organism process	11993	46	34.15	1.50 × 10^−3^
GO:0006067	ethanol metabolic process	1	1	0	2.85 × 10^−3^
GO:0006069	ethanol oxidation	1	1	0	2.85 × 10^−3^
GO:0009233	menaquinone metabolic process	1	1	0	2.85 × 10^−3^
GO:0009234	menaquinone biosynthetic process	1	1	0	2.85 × 10^−3^
GO:0009817	defense response to fungus, incompatible interaction	31	2	0.09	3.52 × 10^−3^
GO:0009814	defense response, incompatible interaction	398	5	1.13	5.48 × 10^−3^
GO:0006522	alanine metabolic process	2	1	0.01	5.69 × 10^−3^
GO:0009078	pyruvate family amino acid metabolic process	2	1	0.01	5.69 × 10^−3^
GO:0018345	protein palmitoylation	2	1	0.01	5.69 × 10^−3^
GO:0006096	glycolytic process	262	4	0.75	6.62 × 10^−3^
GO:0010310	regulation of hydrogen peroxide metabolic process	143	3	0.41	7.91 × 10^−3^
GO:0006103	2-oxoglutarate metabolic process	3	1	0.01	8.52 × 10^−3^
GO:0006531	aspartate metabolic process	3	1	0.01	8.52 × 10^−3^
GO:0090470	shoot organ boundary specification	3	1	0.01	8.52 × 10^−3^
GO:0048856	anatomical structure development	3042	16	8.66	9.66 × 10^−3^

^a^Number of genes mapped to the GO term from all annotated *Musa* genes.

^b^Number of genes mapped to the GO term in the DEGs common to the three genotypes, and

^c^expected number these genes mapped to the GO term if they were randomly distributed over all GO terms.

**Table 4:** List of the 20 candidate genes up-regulated in root during mild osmotic stress and selected for RT-qPCR validation.

Gene ID^a^	FC *(p*-value)^b^			*Arabidopsis thaliana*ortholog(s)	Function^c^	*Musa*family ID^d^(No. paralogs)
	Cach	GN	Mbw			
*GSMUA_Achr**1T23550**_001*	**2.0 × 10**^**1**^(1** × **10^−5^)	**4.0 × 10**^**0**^(2** × **10^−2^)#	**6.3 × 10**^**0**^(4** × **10^−3^)#	*AT2G19350 AT4G29850*	Unknown function (DUF872)	CF158574 (4)
*GSMUA_Achr**1T27360**_001*	**2.9 × 10**^**1**^(2** × **10^−9^)	**6.3 × 10**^**0**^(2** × **10^−3^)#	**5.0 × 10**^**0**^(6** × **10^−3^)#	*AT1G53580*	Mononuclear Fe(II)-containing member of the b-lactamase fold superfamily (ETHE1-like)	CF158578 (2)
*GSMUA_Achr**2T02480**_001*	**3.4 × 10**^**3**^(2** × **10^−12^)	**3.7 × 10**^**3**^(9** × **10^−17^)	**2.5 × 10**^**2**^(3** × **10^−7^)	*AT1G03475*	Coproporphyrinogen III oxidase	CF104144 (3)
*GSMUA_Achr**2T07320**_001*	**3.1 × 10**^**1**^(9** × **10^−9^)	**1.2 × 10**^**1**^(2** × **10^−5^)	**2.0 × 10**^**1**^(2** × **10^−7^)	*AT1G17290 AT1G72330*	Alanine aminotransferase (AlaAT)	CF104167 (3)
GSMUA_Achr**2T08040**_001*	**1.0 × 10**^**1**^(1** × **10^−4^)	**5.6 × 10**^**0**^(2** × **10^−2^)#	**2.1 × 10**^**1**^(8** × **10^−7^)	*AT1G77120*	Alcohol dehydrogenase (ADH)	CF104189 (5)
*GSMUA_Achr**2T08720**_001*	**1.8 × 10**^**2**^(2** × **10^−15^)	**1.5 × 10**^**1**^(3** × **10^−5^)	**3.0 × 10**^**1**^(1** × **10^−6^)	*AT2G16060 AT3G10520*	Class I nonsymbiotic haemoglobin (HB)	CF158581 (3)
*GSMUA_Achr**2T14540**_001*	**2.5 × 10**^**0**^(6** × **10^−2^)#	**2.0 × 10**^**0**^(2** × **10^−1^)#	**2.5 × 10**^**0**^(6** × **10^−2^)	*AT3G18170 AT3G18180*	Glycosyltransferase family 61 protein	CF158591 (5)
GSMUA_Achr**4T08240**_001*	**1.2 × 10**^**1**^(4** × **10^−6^)	**4.0 × 10**^**0**^(2** × **10^−2^)#	**4.0 × 10**^**0**^(1** × **10^−2^)#	*AT4G26270 AT4G29220 AT5G56630 AT4G32840*	6-phosphofructokinase (6PFK)	CF158617 (5)
*GSMUA_Achr**5T16900**_001*	**9.1 × 10**^**2**^(4** × **10^−8^)	**3.0 × 10**^**3**^(1** × **10^−9^)	**6.8 × 10**^**1**^(1** × **10^−5^)	*AT4G10040 AT1G22840*	Cytochrome c (CYTC)	CF158619 (5)
*GSMUA_Achr**5T29560**_001*	**6.5 × 10**^**2**^(9** × **10^−9^)	**1.2 × 10**^**2**^(6** × **10^−10^)	**4.3 × 10**^**2**^(8** × **10^−8^)	*AT5G27760 AT3G05550*	Hypoxia responsive family protein	CF158650 (6)
*GSMUA_Achr**6T04470**_001*	**1.0 × 10**^**3**^(7** × **10^−10^)	**2.1 × 10**^**3**^(6** × **10^−9^)	**9.9 × 10**^**2**^(8** × **10^−6^)	*AT3G28490 AT3G28480*	Prolyl 4-hydroxylase, alpha subunit (P4H)	CF158570 (3)
*GSMUA_Achr**7T03060**_001*	**8.9 × 10**^**1**^(4** × **10^−7^)	**2.9 × 10**^**3**^(9** × **10^−10^)	**6.0 × 10**^**2**^(2** × **10^−5^)	*AT1G43800*	Stearoyl-acyl-carrier-protein desaturase family protein (S-ACP-DES)	CF158628 (2)
*GSMUA_Achr**7T13070**_001*	**2.5 × 10**^**5**^(1** × **10^−26^)	**3.9 × 10**^**5**^(3** × **10^−10^)	**8.1 × 10**^**3**^(8** × **10^−10^)	*AT1G21890 AT1G44800 AT4G08290 AT2G37460*	Nodulin MtN21-like transporter family protein (UMAMIT)	CF104204 (5)
*GSMUA_Achr**8T08600**_001*	**5.0 × 10**^**0**^(3** × **10^−3^)#	**6.3 × 10**^**0**^(7** × **10^−3^)#	**4.0 × 10**^**0**^(9** × **10^−3^)#	*AT5G19140*	Unknown function (DUF3700)	CF158620 (4)
*GSMUA_Achr**8T21350**_001*	**1.8 × 10**^**1**^(7** × **10^−6^)	**1.6 × 10**^**1**^(9** × **10^−7^)	**1.6 × 10**^**1**^(4** × **10^−5^)	*AT3G10920 AT3G56350*	Manganese superoxide dismutase (MSD)	CF158635 (4)
*GSMUA_Achr**8T33540**_001*	**2.7 × 10**^**1**^(4** × **10^−7^)	**5.0 × 10**^**1**^(3** × **10^−9^)	**2.3 × 10**^**1**^(2** × **10^−5^)	*AT5G44730*	Haloacid dehalogenase-like hydrolase (HAD) superfamily protein	CF158624 (2)
GSMUA_Achr**9T23750**_001*	**6.7 × 10**^**1**^(7** × **10^−8^)	**1.4 × 10**^**1**^(1** × **10^−4^)	**1.8 × 10**^**1**^(7** × **10^−5^)	*AT5G08570 AT5G63680*	Pyruvate kinase family protein (PK)	CF158643 (5)
*GSMUA_Achr**9T26700**_001*	**1.0 × 10**^**5**^(1** × **10^−8^)	**4.2 × 10**^**4**^(1** × **10^−10^)	**7.2 × 10**^**3**^(10** × **10^−9^)	*AT3G29970*	B12D protein	CF158632 (2)
GSMUA_Achr**11T24780**_001*	**1.5 × 10**^**2**^(4** × **10^−8^)	**4.7 × 10**^**2**^(1** × **10^−7^)	**5.2 × 10**^**2**^(1** × **10^−8^)	*AT5G01330 AT5G01320 AT4G33070 AT5G54960*	Thiamine pyrophosphate dependent pyruvate decarboxylase family protein (PDC)	CF158639 (6)
*GSMUA_Achr**Un_randomT11830**_001*	**7.9 × 10**^**1**^(1** × **10^−14^)	**4.1 × 10**^**1**^(3** × **10^−9^)	**2.3 × 10**^**1**^(4** × **10^−8^)	*AT2G32720*	Member of Cytochromes b5 (CB5-B)	CF158596 (4)

^a^*Musa* candidate genes (abbreviation in bold) selected from RNA-seq data.

^b^Fold Change (stress vs. control) calculated by edgeR-RLE, and associated *p*-value.

^c^Function of the *A. thaliana* ortholog(s) with the highest sequence similarity to the *Musa* candidate gene (underlined).

^d^Family ID for Custom families created in GreenPhyl database (http://www.greenphyl.org/) and number of *Musa* paralogous genes since divergence with *A. thaliana*. Cach: Cachaco, GN: Grande Naine, Mbw: Mbwazirume.

^*^Candidate genes involved in the glycolysis-fermentation pathway.

^#^not detected at FDR≤0.05, but detected by the non-parametric test combined with PLS.

**Table 5:** ANOVA results showing the significance level of the genotype, treatment and genotype x treatment effects for the 20 candidate genes analysed by RT-qPCR.

Gene ID	Genotype effect	Treatment effect	Genotype × treatment effect
	*p*-value		
*GSMUA_Achr1T23550_001*	***	****	ns
*GSMUA_Achr1T27360_001*	***	****	ns
*GSMUA_Achr2T02480_001*	***	****	ns
*GSMUA_Achr2T07320_001*	*	****	ns
*GSMUA_Achr2T08040_001*	ns	****	ns
*GSMUA_Achr2T08720_001*	ns	****	ns
*GSMUA_Achr2T14540_001*	****	*	ns
*GSMUA_Achr4T08240_001*	*	****	ns
*GSMUA_Achr5T16900_001*	**	****	ns
*GSMUA_Achr5T29560_001*	*	****	ns
*GSMUA_Achr6T04470_001*	*	****	ns
*GSMUA_Achr7T03060_001*	*	****	*
*GSMUA_Achr7T13070_001*	ns	****	ns
*GSMUA_Achr8T08600_001*	ns	**	ns
*GSMUA_Achr8T21350_001*	**	****	ns
*GSMUA_Achr8T33540_001*	***	****	ns
*GSMUA_Achr9T23750_001*	ns	****	ns
*GSMUA_Achr9T26700_001*	***	****	ns
*GSMUA_Achr11T24780_001*	**	****	ns
*GSMUA_AchrUn_randomT11830*	*	****	ns

Genotypes used: Cachaco, Grande Naine and Mbwazirume. Number of biological replicates (stress/control): n = 6/6. *p < 0.05, **p < 0.01, ***p < 0.001, ****p < 0.0001. ns: not significant. Candidate genes involved in the glycolysis-fermentation pathway are underlined.
