# Corrigendum: Differential root transcriptomics in a polyploid non-model crop: the importance of respiration during osmotic stress

**DOI:** 10.1038/srep25683

**Published:** 2016-05-20

**Authors:** Yasmín Zorrilla-Fontanesi, Mathieu Rouard, Alberto Cenci, Ewaut Kissel, Hien Do, Emeric Dubois, Sabine Nidelet, Nicolas Roux, Rony Swennen, Sebastien Christian Carpentier

Scientific Reports 6: Article number: 22583; 10.1038/srep22583 published online: 03032016; updated: 05202016.

In this Article, the Accession codes were omitted from the Additional Information section:

Data availability: The short reads of RNA-seq were deposited in NCBI’s Sequence Read Archive (SRA) database with accession numbers from SRX1473480 to SRX1473497 (http://www.ncbi.nlm.nih.gov/bioproject/PRJNA305241). BAM files were included in Banana Genome Hub and can be visualized with genome browser of the (http://banana-genome-hub.southgreen.fr/jbrowse_ma1).

