# Supplementary material for: Differential root transcriptomics in a polyploid non-model crop: the importance of respiration during osmotic stress
Source: Sci Rep. 2016 Mar 3;6:22583. doi: 10.1038/srep22583 (PMC4776286; doi:10.1038/srep22583)
Supplement: Supplementary figures [file srep22583-s1.doc]

**Supplementary information for following article**

**Differential root transcriptomics in a polyploid non-model crop: the importance of respiration during osmotic stress**.

Yasmin Zorrilla-Fontanesi^1^, Mathieu Rouard^2^, Alberto Cenci^2^, Ewaut Kissel^1^, Hien Do^1^, Emeric Dubois^3^, Sabine Nidelet^3^, Nicolas Roux^2^, Rony Swennen^1,4,5^, Sebastien Christian Carpentier^1*^.

^1^Laboratory of Tropical Crop Improvement, Division of Crop Biotechnics, KU Leuven, B-3001 Leuven, Belgium.

^2^Bioversity International, Parc Scientifique Agropolis II, 34397 Montpellier Cedex 05, France.

^3^MGX-Montpellier GenomiX, Montpellier Genomics and Bioinformatics Facility, Montpellier F-34396, France.

^4^Bioversity International, Willem De Croylaan 42, B-3001 Leuven, Belgium.

^5^International Institute of Tropical Agriculture. c/o AVRDC - The World Vegetable Center. P.O. Box 10, Duluti, Arusha, Tanzania.

Yasmin Zorrilla-Fontanesi^1^ and Mathieu Rouard^2^ contributed equally to this work.

*Corresponding author:

Laboratory of Tropical Crop Improvement, Division of Crop Biotechnics, KU Leuven, B-3001 Leuven, Belgium.

Sebastien Christian Carpentier.

E-mail: [Sebastien.Carpentier@kuleuven.be](mailto:Sebastien.Carpentier@kuleuven.be)

Tel:+32 16 3 79311 or +32 16 3 21421

**
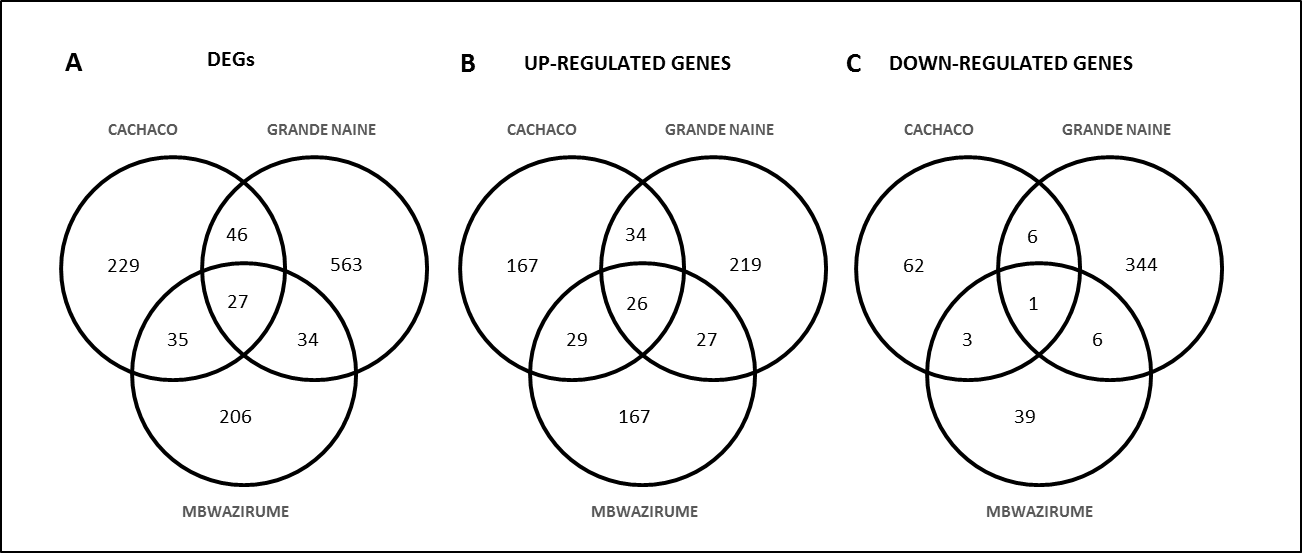
**

**Supplementary Figure S1. Venn diagrams illustrating the number of differentially expressed, up- and down-regulated genes detected by edgeR-RLE (FDR≤0.05) in root after 3 days of mild osmotic stress.** Genotype comparison of specificity and commonality of **A)** differentially expressed genes (DEGs), **B)** up-regulated genes, and **C)** down-regulated genes. Number of biological replicates (stress/control): n=3/3; FDR: false discovery rate.

**
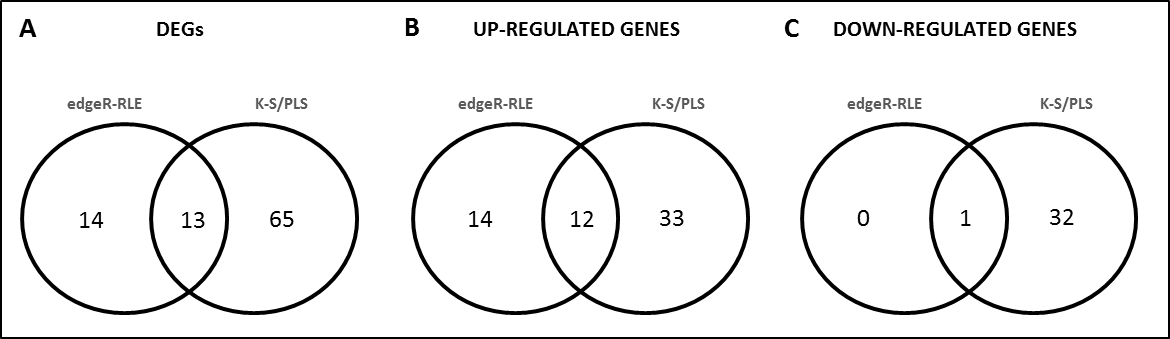
**

**Supplementary Figure S2. Venn diagram comparison of differentially expressed, up- and down-regulated genes commonly detected in all three genotypes by edgeR-RLE (FDR≤0.05) and/or non-parametrics Kolmogorov-Smirnov test (p≤0.1) combined with PLS.** Number of **A)** differentially expressed genes; **B)** up-regulated genes; **C)** down-regulated genes. Number of biological replicates (stress/control): n=3/3; DEGs: differentially expressed genes; FDR: false discovery rate; PLS: Partial Least Squares. K-S: Kolmogorov-Smirnov test.

**
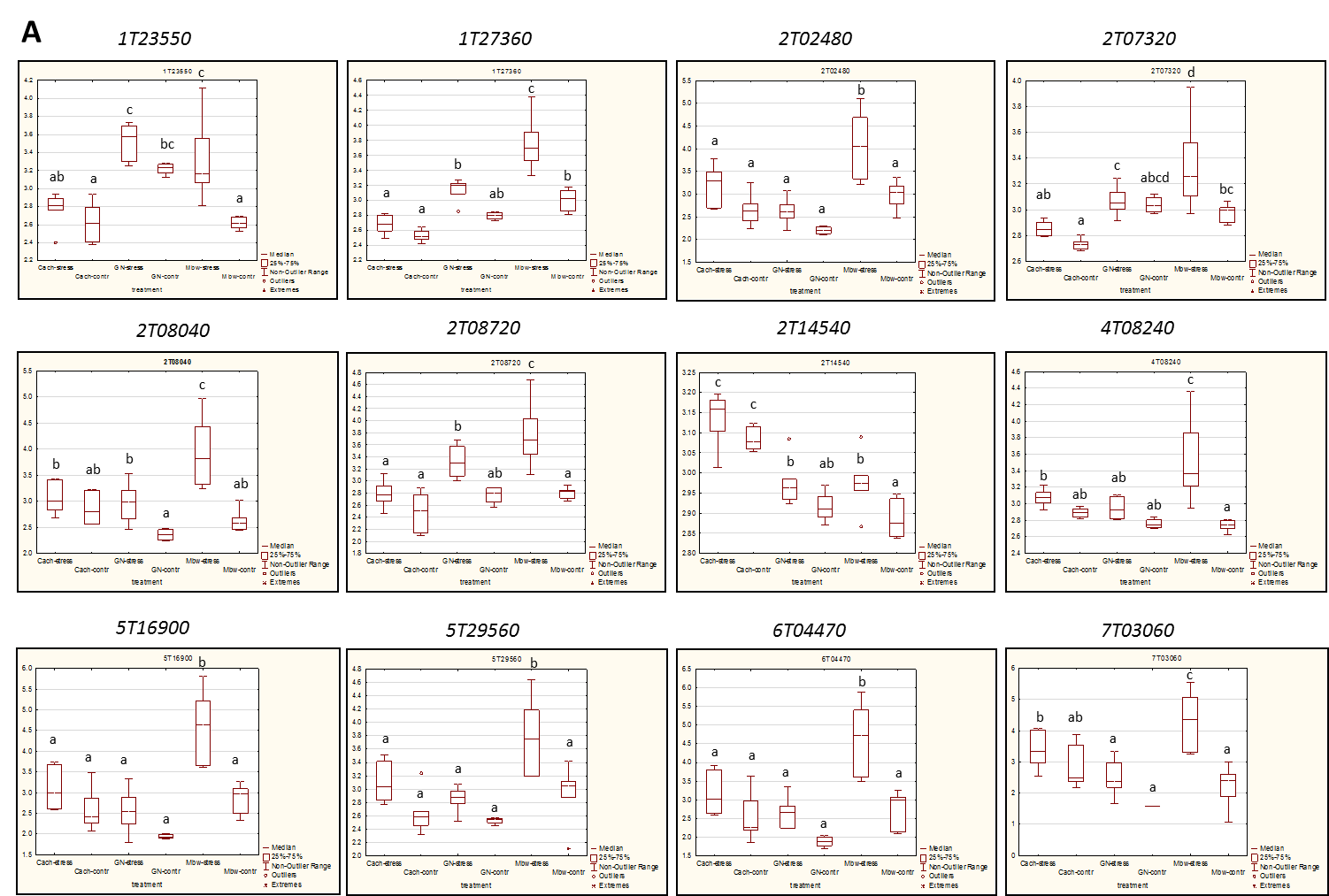
**

**Supplementary Figure S3. Boxplots showing the relative expression levels of the 20 candidate genes validated by RT-qPCR.** Expression levels after **A)** 6 hours of 5% PEG treatment, **B)** 3 days of 5% PEG treatment, **C)** 7 days of 5% PEG treatment. X-axis: genotype and treatment groups (Cach: Cachaco; GN: Grande Naine; Mbw: Mbwazirume). Group mean comparisons by Fisher’s LSD test (a˂b˂c˂d˂e). Y-axis: relative expression values in log10 scale. Gene ID abbreviations according to Table 3.

**
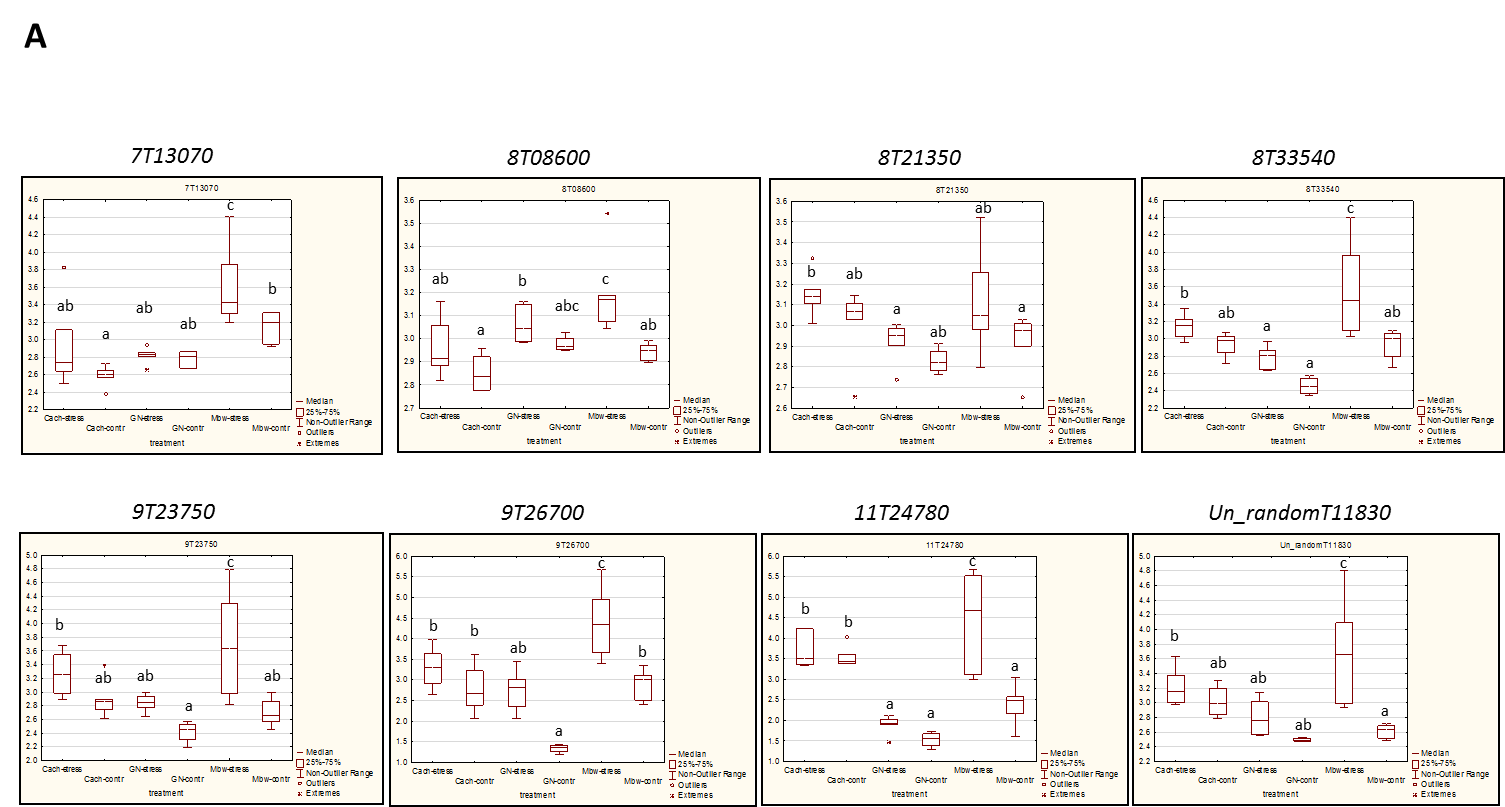
**

**Supplementary Figure S3.** Continued.

**
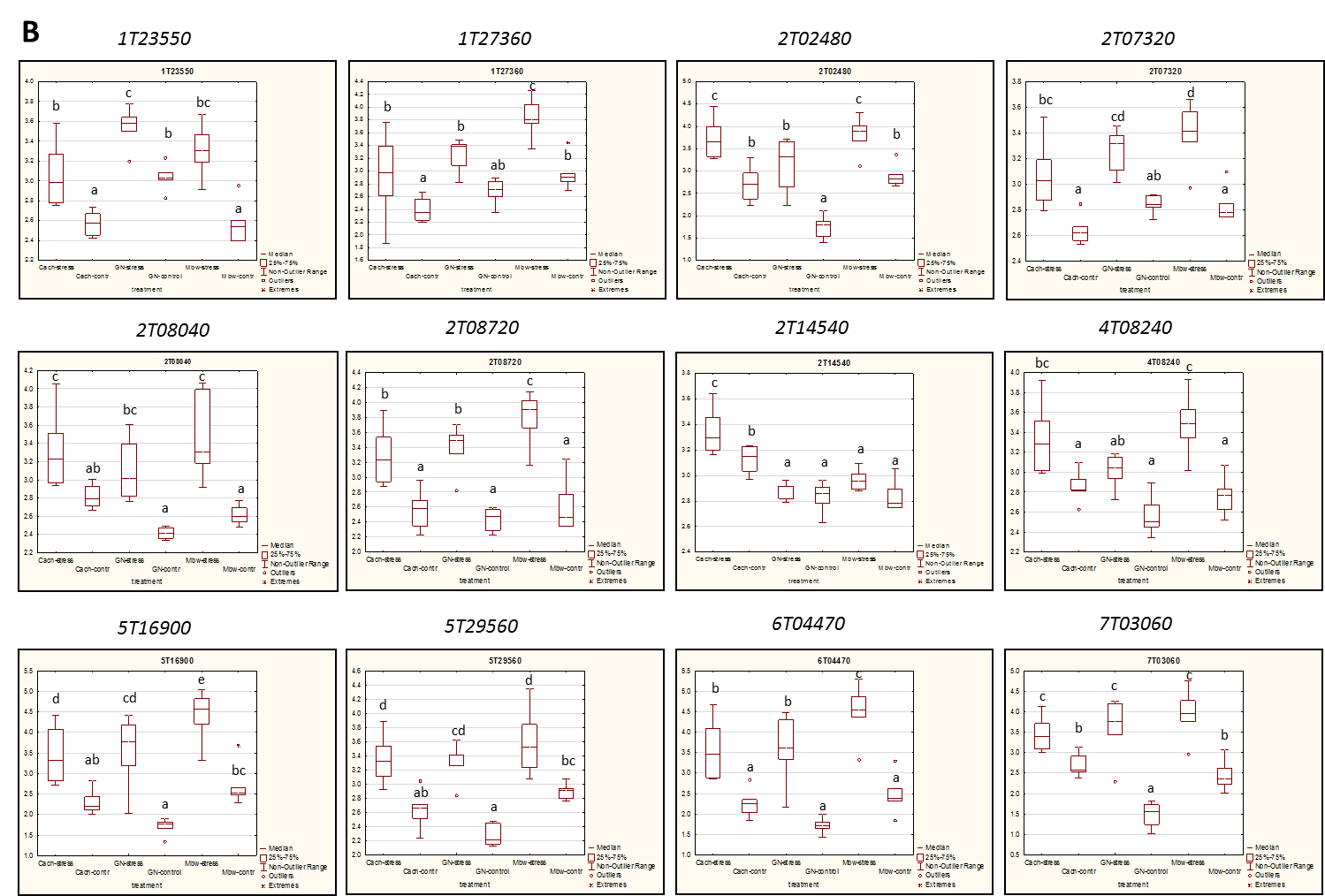
**

**Supplementary Figure S3.** Continued.

**
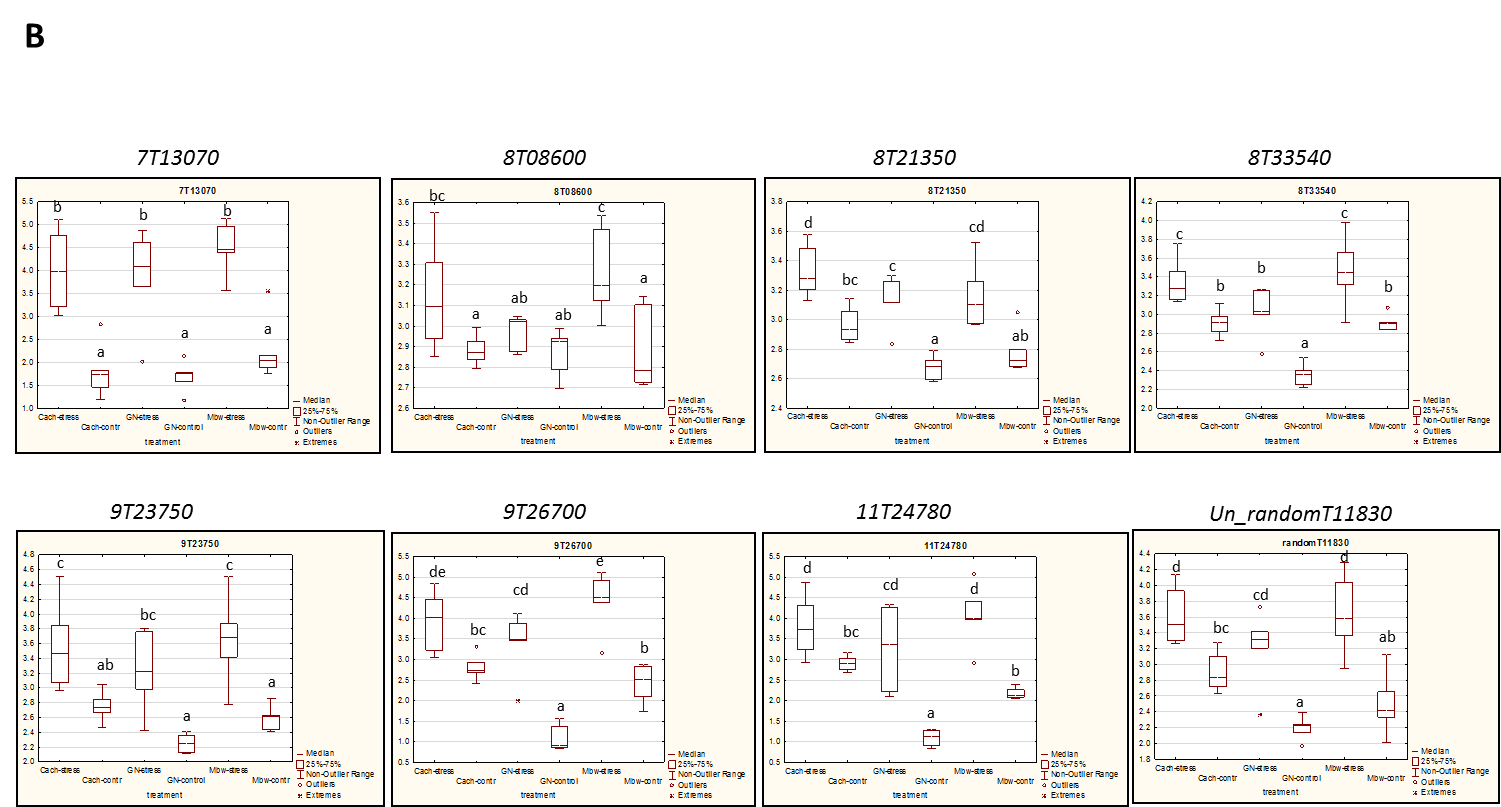
**

**Supplementary Figure S3.** Continued.

**
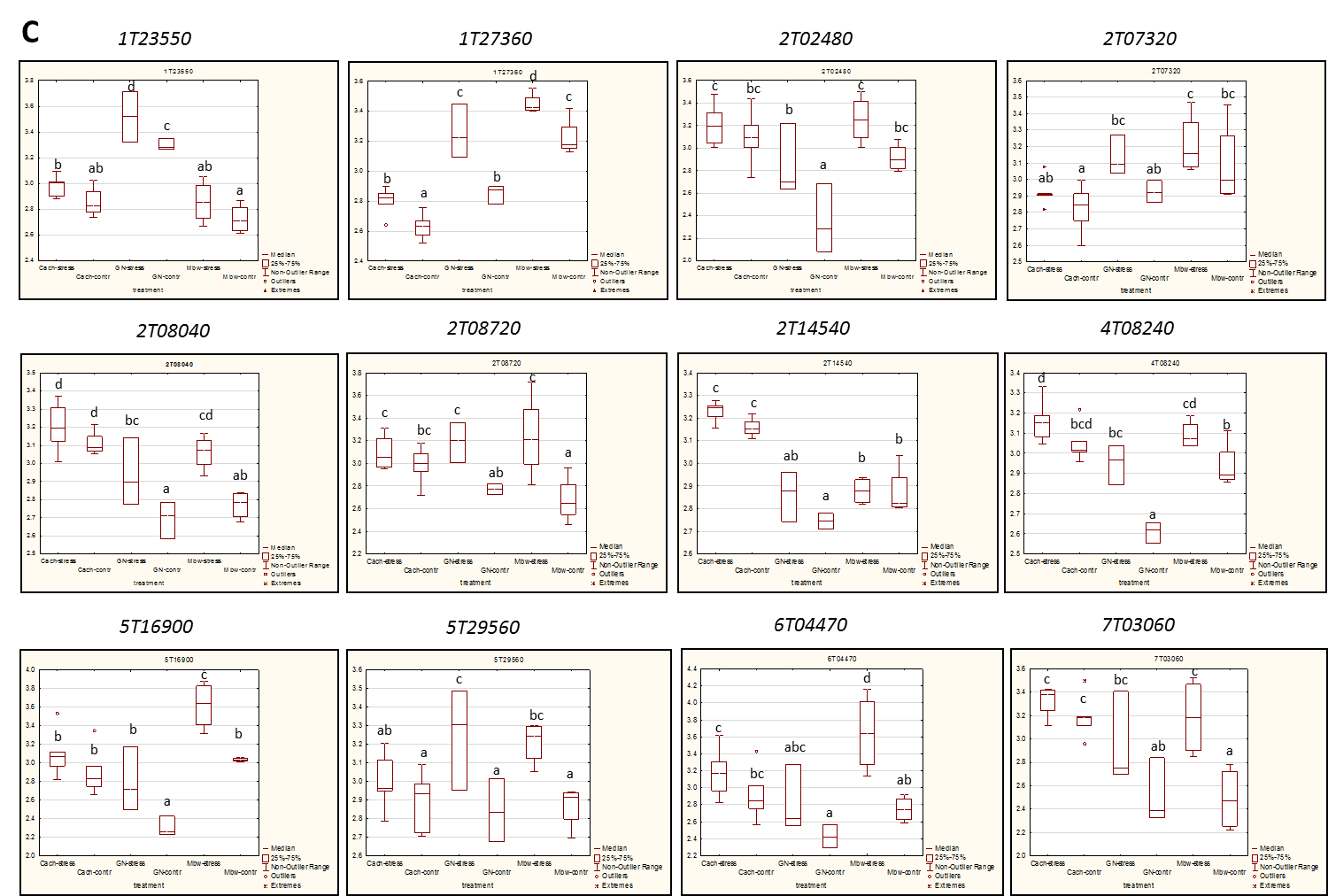
**

**Supplementary Figure S3.** Continued.

**
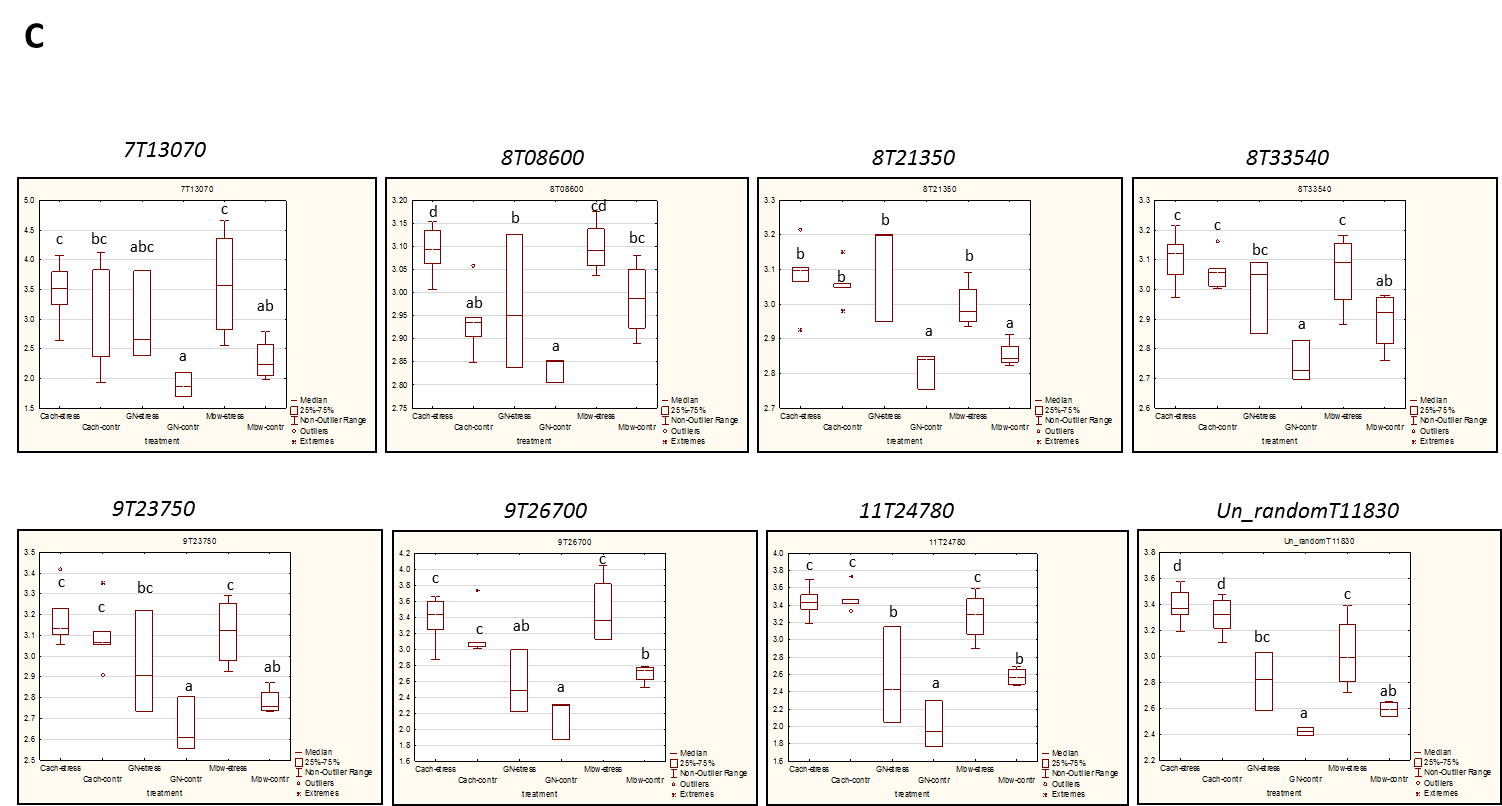
**

**Supplementary Figure S3.** Continued.

**
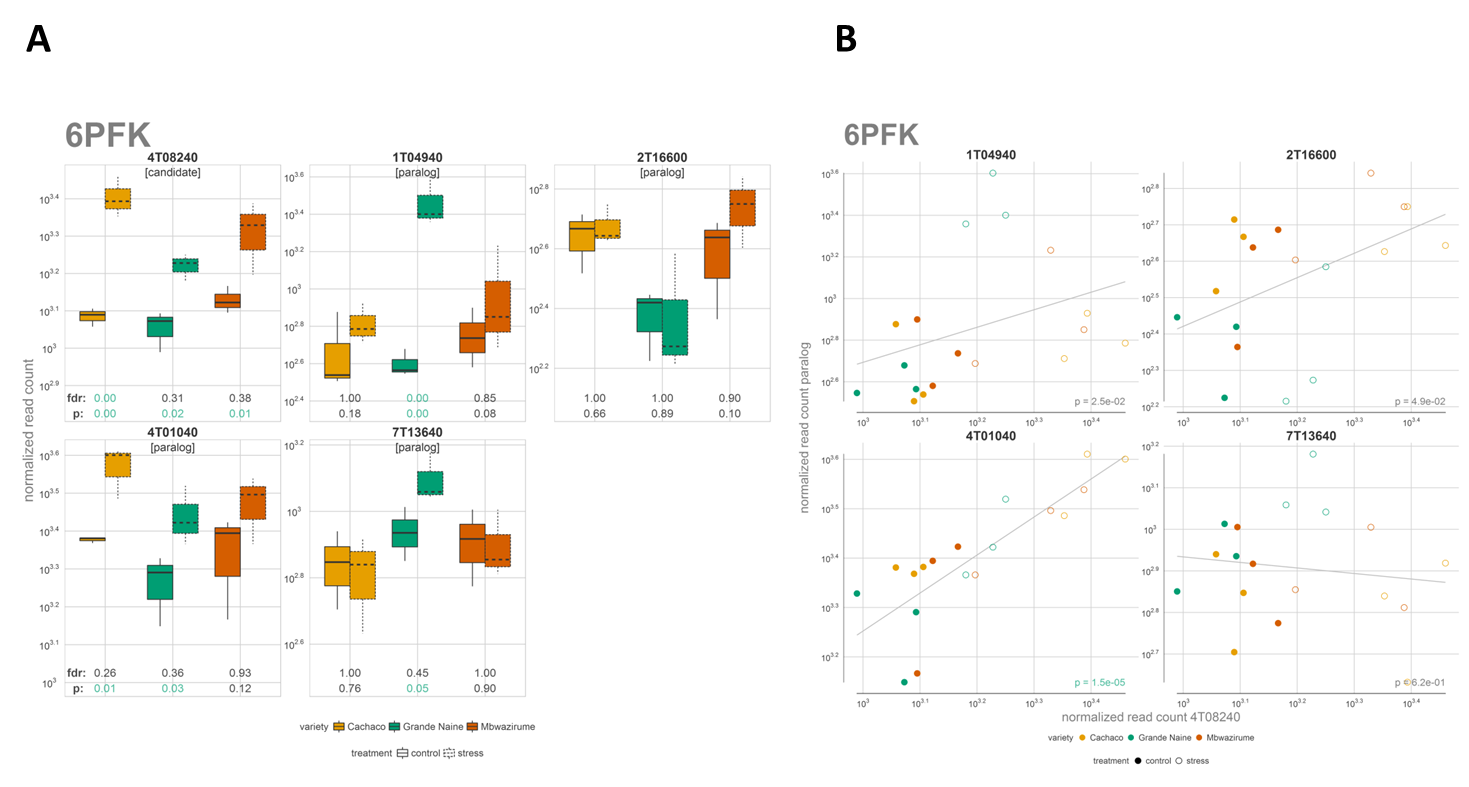
**

**Supplementary Figure S4. Expression patterns of the candidate genes involved in glycolysis-fermentation and their corresponding paralogs. A)** Boxplots showing the expression levels for each cultivar/treatment combination and significance level of the edgeR-RLE test. Outlier range is indicated as 0.25 - 1.5 IQR and 0.75 + 1.5 IQR. **B)** Spearman rank correlations between the candidate gene (x-axis) and the corresponding paralogs (y-axis). Number of biological replicates (stress/control): n=3/3. IQR: interquartile range. fdr: false discovery rate. p = p-value. Significant results are highlighted in green. 6PFK: 6-phosphofructokinase. PK: pyruvate kinase. PDC: pyruvate decarboxylase. ADH: alcohol dehydrogenase. Gene ID abbreviations according to Table 3. Only paralogs with expression in root are plotted.

**
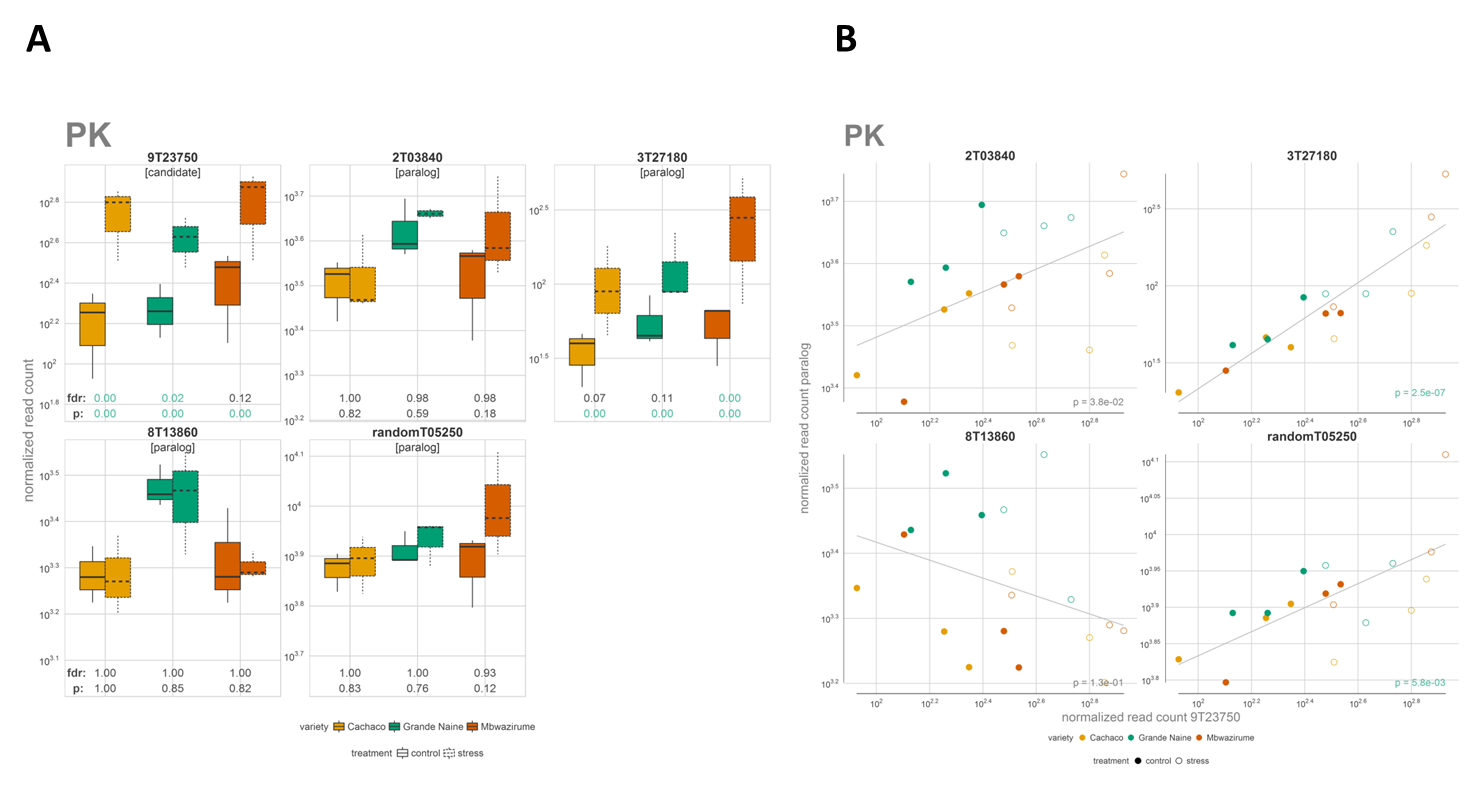
**

**Figure S4.** Continued.

**
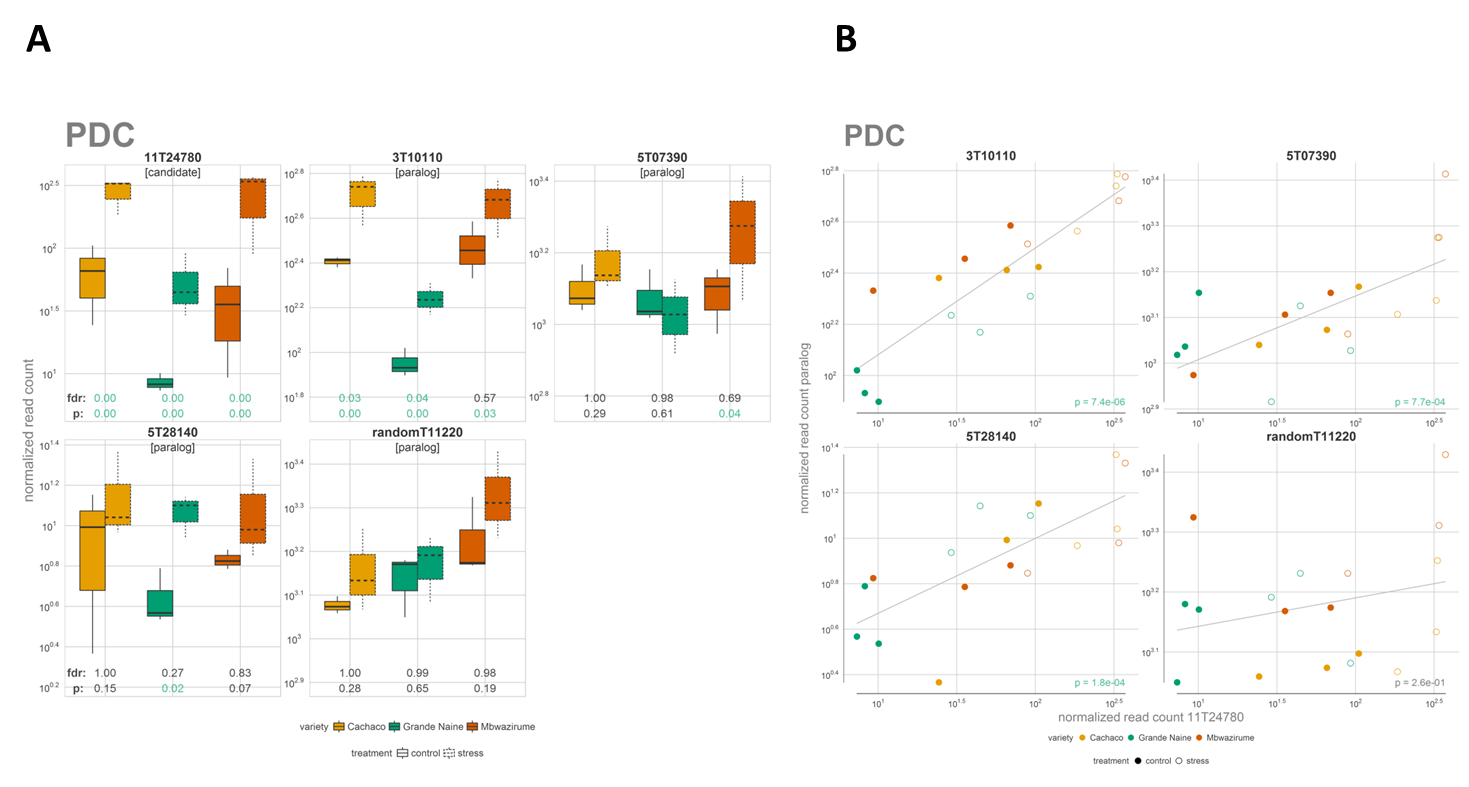
**

**Figure S4.** Continued.

**
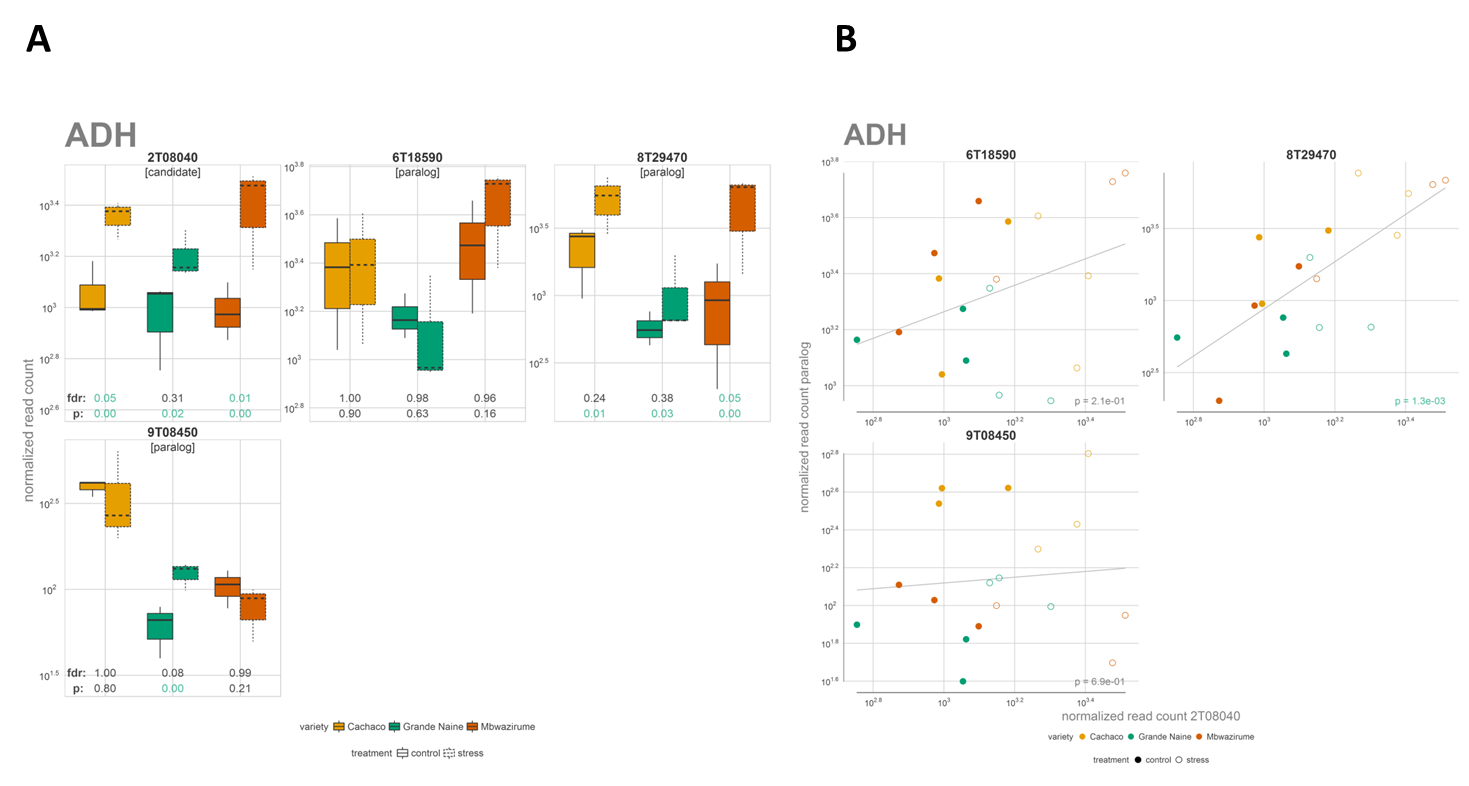
**

**Figure S4.** Continued.

**
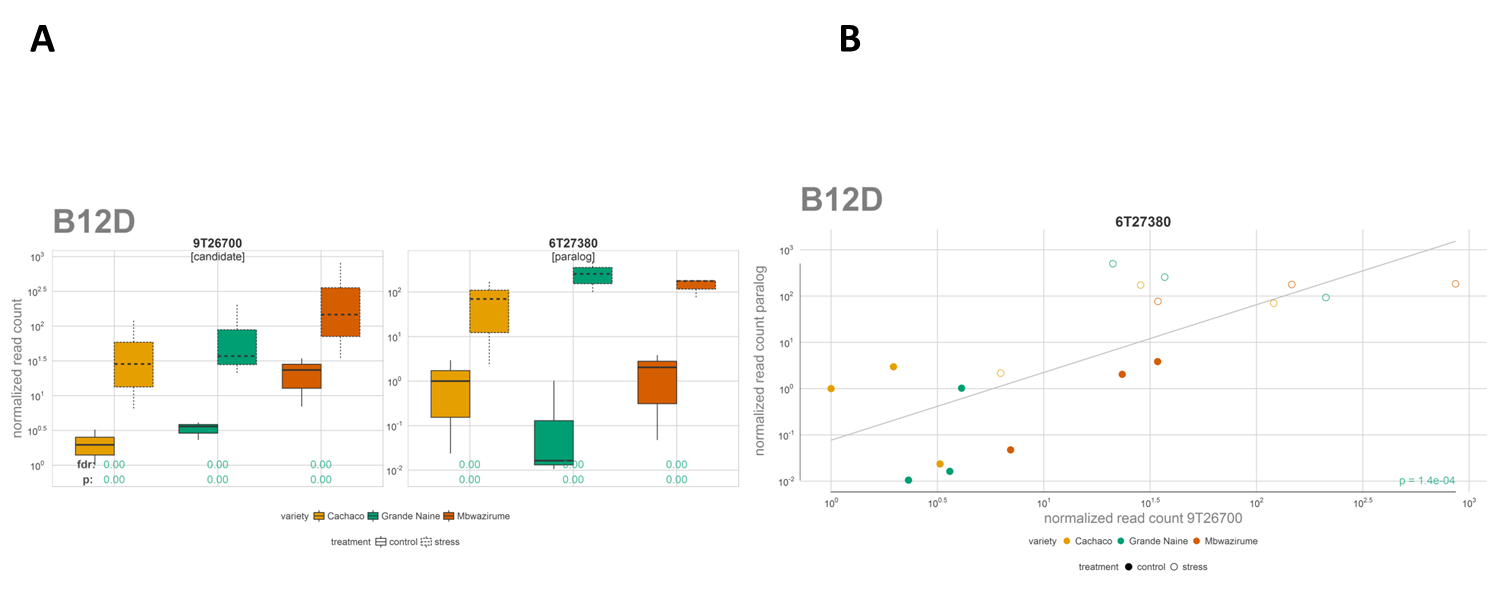
**

**Supplementary Figure S5. Expression patterns of the B12D candidate gene and corresponding paralog. A)** Boxplots showing the expression levels for each cultivar/treatment combination and significance level of the edgeR-RLE test. Outlier range is indicated as 0.25 - 1.5 IQR and 0.75 + 1.5 IQR. **B)** Spearman rank correlations between the candidate gene (x-axis) and the corresponding paralog (y-axis). Number of biological replicates (stress/control): n=3/3. IQR: interquartile range. fdr: false discovery rate. p = p-value. Significant results are highlighted in green. Gene ID abbreviations according to Table 3.

**
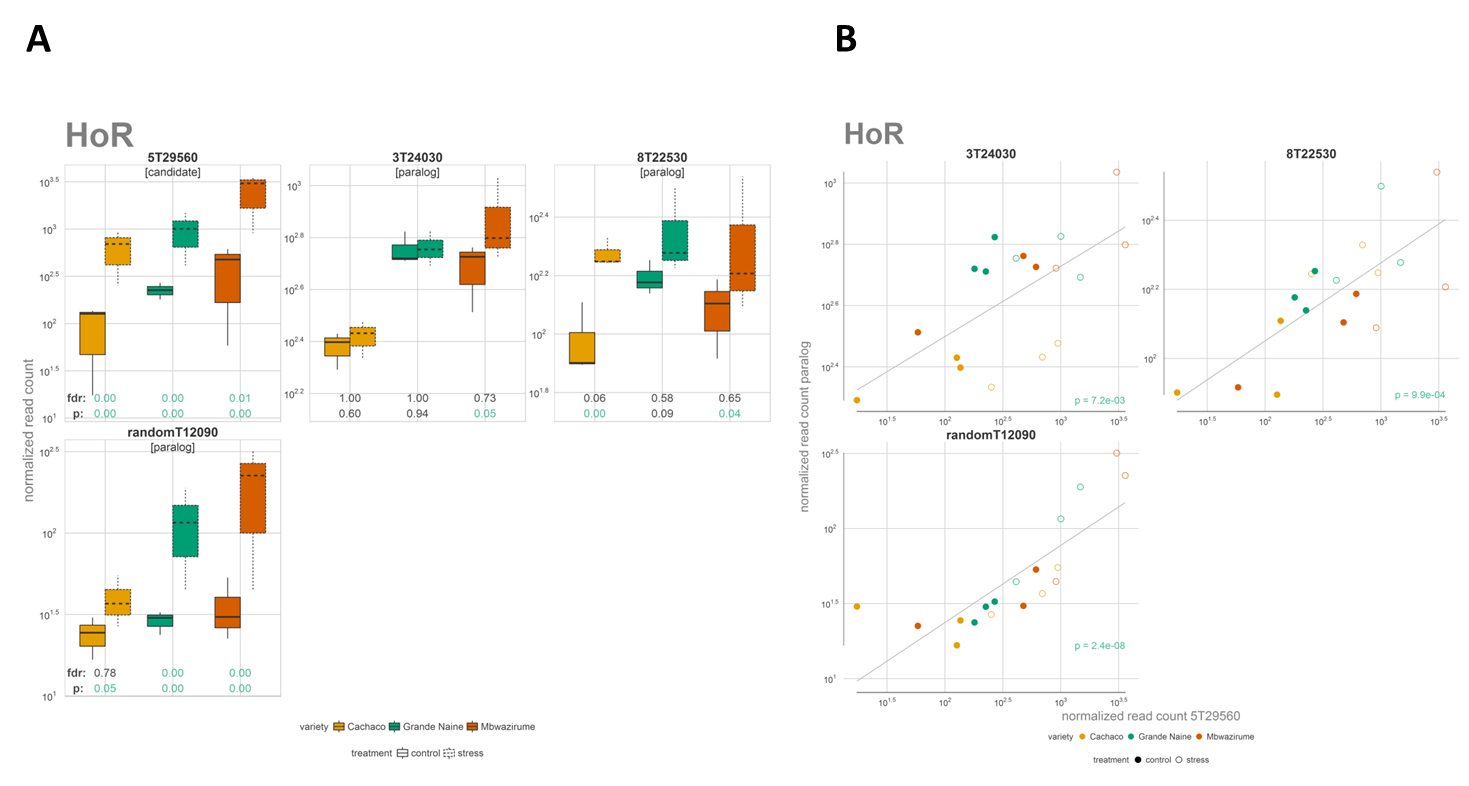
**

**Supplementary Figure S6. Expression patterns of the hypoxia responsive candidate gene and corresponding paralogs. A)** Boxplots showing the expression levels for each cultivar/treatment combination and significance level of the edgeR-RLE test. Outlier range is indicated as 0.25 - 1.5 IQR and 0.75 + 1.5 IQR. **B)** Spearman rank correlations between the candidate gene (x-axis) and the corresponding paralogs (y-axis). Number of biological replicates (stress/control): n=3/3. IQR: interquartile range. fdr: false discovery rate. p = p-value. Significant results are highlighted in green. HoR: hypoxia responsive family protein. Gene ID abbreviations according to Table 3. Only paralogs with expression in root and correct annotation are plotted.

**
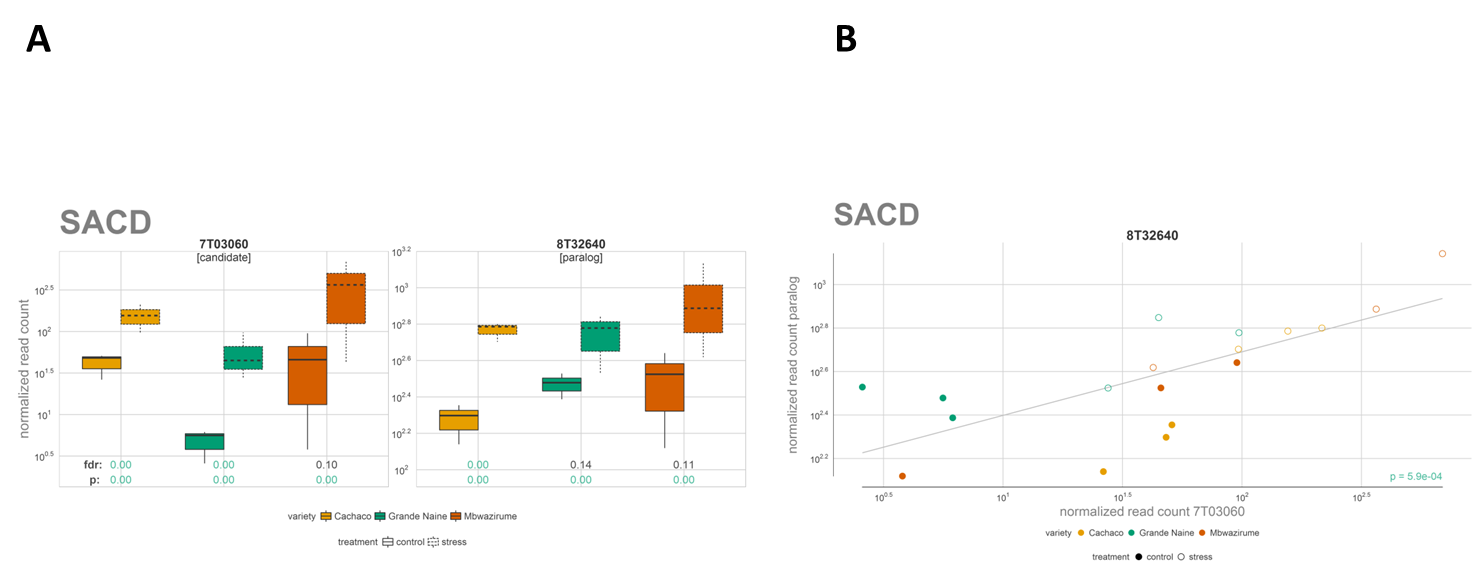
**

**Supplementary Figure S7. Expression patterns of the stearoyl-acyl-carrier-protein desaturase candidate gene and corresponding paralog. A)** Boxplots showing the expression levels for each cultivar/treatment combination and significance level of the edgeR-RLE test. Outlier range is indicated as 0.25 - 1.5 IQR and 0.75 + 1.5 IQR. **B)** Spearman rank correlations between the candidate gene (x-axis) and the corresponding paralog (y-axis). Number of biological replicates (stress/control): n=3/3. IQR: interquartile range. fdr: false discovery rate. p = p-value. Significant results are highlighted in green. SACD: stearoyl-acyl-carrier-protein desaturase family protein. Gene ID abbreviations according to Table 3.
